# Clmp Regulates AMPA and Kainate Receptor Responses in the Neonatal Hippocampal CA3 and Kainate Seizure Susceptibility in Mice

**DOI:** 10.3389/fnsyn.2020.567075

**Published:** 2020-12-21

**Authors:** Seil Jang, Esther Yang, Doyoun Kim, Hyun Kim, Eunjoon Kim

**Affiliations:** ^1^Center for Synaptic Brain Dysfunctions, Institute for Basic Science, Daejeon, South Korea; ^2^Department of Anatomy and Division of Brain Korea 21, Biomedical Science, College of Medicine, Korea University, Seoul, South Korea; ^3^Center for Drug Discovery Platform Research, Korea Research Institute of Chemical Technology (KRICT), Daejeon, South Korea; ^4^Department of Biological Sciences, Korea Advanced Institute of Science and Technology (KAIST), Daejeon, South Korea

**Keywords:** synaptic adhesion molecule, synaptic transmission, AMPA receptors, kainate receptors, NMDA receptors, hippocampus, learning and memory, seizure

## Abstract

Synaptic adhesion molecules regulate synapse development through trans-synaptic adhesion and assembly of diverse synaptic proteins. Many synaptic adhesion molecules positively regulate synapse development; some, however, exert negative regulation, although such cases are relatively rare. In addition, synaptic adhesion molecules regulate the amplitude of post-synaptic receptor responses, but whether adhesion molecules can regulate the kinetic properties of post-synaptic receptors remains unclear. Here we report that Clmp, a homophilic adhesion molecule of the Ig domain superfamily that is abundantly expressed in the brain, reaches peak expression at a neonatal stage (week 1) and associates with subunits of AMPA receptors (AMPARs) and kainate receptors (KARs). *Clmp* deletion in mice increased the frequency and amplitude of AMPAR-mediated miniature excitatory post-synaptic currents (mEPSCs) and the frequency, amplitude, and decay time constant of KAR-mediated mEPSCs in hippocampal CA3 neurons. *Clmp* deletion had minimal impacts on evoked excitatory synaptic currents at mossy fiber-CA3 synapses but increased extrasynaptic KAR, but not AMPAR, currents, suggesting that Clmp distinctly inhibits AMPAR and KAR responses. Behaviorally, *Clmp* deletion enhanced novel object recognition and susceptibility to kainate-induced seizures, without affecting contextual or auditory cued fear conditioning or pattern completion-based contextual fear conditioning. These results suggest that Clmp negatively regulates hippocampal excitatory synapse development and AMPAR and KAR responses in the neonatal hippocampal CA3 as well as object recognition and kainate seizure susceptibility in mice.

## Introduction

Synaptic adhesion molecules mediate and regulate diverse aspects of synapse development and function (Dalva et al., 2007; Shen and Scheiffele, 2010; Siddiqui and Craig, 2011; Takahashi and Craig, 2013; Um and Ko, 2013; Ko et al., 2015; de Wit and Ghosh, 2016; Jang et al., 2017; Krueger-Burg et al., 2017; Südhof, 2017; Sudhof, 2018; Yuzaki, 2018; Kurshan and Shen, 2019; Ribic and Biederer, 2019). Synaptic adhesion molecules localized to early axo-dendritic contacts contribute to synapse maturation by promoting the recruitment and stabilization of various pre- and post-synaptic membrane and cytoplasmic proteins.

Among the important synaptic proteins that are recruited to early synapses by synaptic adhesion molecules are post-synaptic neurotransmitter receptors. Recent studies have reported several such interactions involving, for instance, NMDA (N-methyl-d-aspartate)- and AMPA (α-amino-3-hydroxy-5-methyl-4-isoxazolepropionic acid)-type glutamate receptors (NMDARs and AMPARs) (Nuriya and Huganir, 2006; Saglietti, 2007; Uemura et al., 2010; Zhang et al., 2010; Pozo et al., 2012; Sarto-Jackson et al., 2012; Budreck, 2013; Tomioka et al., 2014; Matsuda et al., 2016; Um et al., 2018). Although detailed mechanisms of these interactions still remain to be clarified, these results suggest that synaptic adhesion molecules and post-synaptic receptors form physical and functional complexes that modulate post-synaptic receptor responses.

AMPARs and kainate receptors (KARs) are members of the ionotropic glutamate receptor (iGluR) family that act as ligand-gated ion channels to mediate excitatory synaptic transmission and plasticity. Both receptor types are grouped together to form the non-NMDAR family (Mayer and Westbrook, 1987; Collingridge and Lester, 1989). Whereas AMPARs mediate fast excitatory synaptic transmission (Huganir and Nicoll, 2013), KARs mediate slow excitatory synaptic transmission (Castillo et al., 1997b). KARs, which are structurally similar to AMPARs (Chen et al., 2003; Nanao et al., 2005), are widely distributed in different brain regions and are highly enriched on the post-synaptic side of mossy fiber (MF)-pyramidal cell synapses in the CA3 region of the hippocampus (Bahn et al., 1994; Castillo et al., 1997b; Bannister et al., 2005; Zhuo, 2017). Exogenous kainate, the agonist of KARs, produces partial, but non-desensitizing, openings of AMPAR channels (Tomita et al., 2007). Importantly, both AMPARs and KARs have been implicated in seizure pathophysiology and have been suggested as potential therapeutic targets (Rawls et al., 2009; Hibi et al., 2012; Kato et al., 2016; Falcon-Moya et al., 2018).

Clmp (CXADR-like membrane protein), also known as ASAM or ACAM, was originally identified as a novel member of the CAR subgroup of the CTX family (Raschperger et al., 2004), and was termed ASAM or ACAM for its expression in adipocytes (Eguchi et al., 2005). Clmp is also expressed in the intestine and is required for intestinal development (van der Werf et al., 2013; Langhorst et al., 2018). Like other members of the CAR subgroup (CAR, ESAM, and IgSF11), Clmp mediates homophilic adhesion (Raschperger et al., 2004; Eguchi et al., 2005). In the human brain, expression of the *CLMP* gene was reported in the developing cerebral neocortex and other brain areas, including the hippocampus, striatum, amygdala, thalamus, and cerebellum (Kang et al., 2011; Pletikos et al., 2014). Recently, members of the CAR subgroup have been shown to regulate synaptic function in the brain. For example, IgSF11 is a synaptic cell-adhesion molecule that interacts with AMPARs and regulates AMPAR-mediated synaptic transmission and plasticity (Jang et al., 2016). CAR, detected in native AMPAR complexes as an AMPAR-interacting membrane protein using shotgun liquid chromatography-tandem mass spectrometry (LC-MS/MS) protein analysis (AP-MS/MS) (Shanks et al., 2012), was found to negatively regulate excitatory synaptic transmission through presynaptic exocytosis-related mechanisms (Wrackmeyer et al., 2019). These results suggest the possibility that Clmp might also regulate aspects of synapse development and function in the brain.

In the present study, we found that Clmp exhibits early post-natal expression and interacts with AMPAR and KAR subunits. Moreover, results from mice lacking Clmp suggest that Clmp negatively regulates synapse development and distinctly suppress AMPAR and KAR responses in the CA3 region of the hippocampus. Behaviorally, *Clmp* deletion also altered object recognition and kainate seizure susceptibility, but not contextual or cued fear conditioning or pattern completion.

## Materials and Methods

### cDNA Constructs

Full-length human Clmp (NM_024769, aa 1–373, Origene RC203362) were amplified by PCR, and subcloned into pGW1-CMV (British Biotechnology). For HA-Clmp, the HA epitope was added to the N-terminus of human Clmp in pGW1. Deletion variants of Clmp were generated by PCR using HA-Clmp (human) as a template and deleting the following regions; aa 317–373 (Clmp ECD-TM), aa 231–373 (Clmp ECD-PDGFR TM), aa 1–316 (Clmp ICD-PDGFR TM). For Clmp ECD-PDGFR TM and Clmp ICD-PDGFR TM, the signal peptide and the transmembrane domain of Clmp were replaced with the N-terminal signal peptide and the C-terminal transmembrane anchoring domain of platelet-derived growth factor receptor (PDGFR) of pDisplay (Invitrogen). All Clmp expression constructs were generated by subcloning the inserts to pGW1 except Clmp ECD-PDGFR was subcloned into pDisplay. The following constructs have been described: pGW1-PSD-95 (Kim et al., 1995). pRK5-GluA1 and pRK5-GluA2 were kindly provided by Dr. Richard Huganir (Shen et al., 2000).

### Antibodies

Peptides containing mouse Clmp (aa 345–373) were used to immunize guinea pigs (2090). The specificity of anti-Clmp antibodies (2090) was confirmed by immunoblot experiments using *Clmp*^−/−^ whole-brain lysates. The following antibodies have been described: PSD-95 (1688) (Yang, 2011), GluA1 (1193) (Kim, 2009), GluA2 (1195) (Kim, 2009). The following antibodies were purchased: HA rabbit polyclonal (Santa Cruz sc-805), HA mouse monoclonal (Boehringer Mannheim 12CA5), PSD-95 (75-028) (NeuroMab), Synaptophysin (Santa Cruz sc-9116), GluA1 (Sigma-Aldrich MAB2263), GluA2 (Sigma-Aldrich MAB397), GluK2 (Sigma-Aldrich 04-921), GluK5 (Sigma-Aldrich 06-315), α-tubulin (Sigma T5168), β-actin (Sigma, A5316).

### Radioisotope *in situ* Hybridization

Mouse brain sections (14 μm thick) at embryonic day (E18) and post-natal days (P1, P7, P14, P21, and P56) were prepared using a cryostat (Leica CM 1950). Hybridization probes specific for mouse Clmp mRNAs were prepared using the following regions: nt 1351–1650 (C-term) of Clmp (NM_133733.4). Antisense riboprobes were generated using 35^S^-uridine triphosphate (UTP) and the Riboprobe system (Promega).

### Fluorescence *in situ* Hybridization

Frozen mouse brain sections (14 μm thick) at post-natal days (P7 and P56) were cut coronally through the hippocampal formation. The sections were fixed in 4% paraform aldehyde for 10 min, dehydrated in increasing concentrations of ethanol for 5 min, and finally air-dried. Tissues were then pre-treated for protease digestion for 10 min at room temperature. The probes used in this study were three synthetic oligonucleotides complementary to the nucleotide (nt) sequence 396–1462 of Mm-Clmp-C1, nt 464–1415 of MmSlc17a7/Vglut1-C2, nt 1986–2998 of Mm-Slc17a6/Vglut2-C3, nt 62–3113 of Mm-Gad1-C3, nt 552–1506 of Mm-Gad2-C2 (ACDBio, Newark, CA, United States). The labeled probes were conjugated to Atto 550 (C1), Alexa Fluor 488 (C2), and Atto 647 (C3). The sections were hybridized at 40°C with labeled probe mixtures (C1 + C2 + C3) per slide for 2 h. Amplification steps involved sequential incubations with Amplifier 1-FL for 30 min, Amplifier 2-FL for 15 min, Amplifier 3-FL for 30 min, and Amplifier 4 Alt B-FL at 40°C for 15 min. Fluorescent images were acquired using TCS SP8 Dichroic/CS (Leica), and the ImageJ program (NIH) was used to analyze the images.

### Brain Lysates Preparation

Whole mouse or rat brain lysates [1-week-old (P7) or 6-week-old (P42)] were prepared as previously described (Jang et al., 2016). After the brain dissection, obtained brain tissues were briefly homogenized in 10 volumes of ice-cold homogenization buffer (0.32 M sucrose, 10 mM HEPES pH 7.4, 2 mM EDTA, protease inhibitors, phosphatase inhibitors). Protein concentrations were measured by the Bradford assay. The relative amount of α-tubulin or β-actin was used as a loading control.

### Subcellular and PSD Fractions

Subcellular and PSD fractions of rat brains [1-week-old (P7)] were prepared as described previously (Jang et al., 2016). Triton X-100-soluble fractions enriched with perisynaptic, presynaptic and extrasynaptic proteins were collected as the non-post-synaptic density membrane fraction (non-PSD). The non-PSD enrichment was checked by PSD-95 and synaptophysin immunoblotting. Immunoblot analysis of these fractions was performed using Clmp (2090), PSD-95 (1688), synaptophysin, α-tubulin, and β-actin antibodies.

### Cell-Surface Biotinylation Assay

Hippocampal slices were prepared as described for electrophysiology. After 1 h, slices were transferred into 24-well plates containing 0.5 mg/ml sulfo-NHS-LC-Biotin (21335; Pierce). Slices were biotinylated for 30 min on ice, followed by three 10-min washes in cold ACSF and then two 25-min washes in ACSF containing 100 mM glycine. The hippocampus was then dissected from each slice and solubilized in RIPA buffer containing 50 mM Tris-HCl, 150 mM NaCl, 1% NP-40, 1% sodium deoxycholate, 2 mM EDTA, supplemented with protease inhibitors for 1 h at 4 °C. Samples were then cleared by centrifugation at 20,000 × g for 30 min and then the supernatants were collected. The samples were then incubated with 40 μl of washed neutravidin agarose beads (29200; Pierce) overnight at 4°C. Beads were then washed three times with lysis buffer, and proteins were eluted by heating at 95°C for 5 min with Laemmli sample buffer containing β-mercaptoethanol. The surface protein enrichment was checked by β-actin immunoblotting.

### Co-immunoprecipitation

HEK293T cells were harvested 36–48 h after transfection and were solubilized in Tris-buffered saline (pH 7.4) containing 50 mM Tris-HCl, 150 mM NaCl, 1% NP-40, 1% sodium deoxycholate, 2 mM EDTA, supplemented with protease inhibitors at 4°C. Following centrifugation at 20,000 × g for 30 min at 4°C, the clarified lysates were subjected to immunoprecipitation using HA monoclonal antibodies coupled to agarose beads (Pierce) for 2 h at 4°C. Following 4–6 washes with 1 ml of the solubilizing buffer, bound proteins were eluted with SDS sample buffer containing 5% β-mercaptoethanol and boiled for 5–10 min for SDS-PAGE analyses.

### Molecular Modeling

The structure of mouse Clmp was generated by homology modeling using the I-TASSER server (Zhang, 2008; Yang and Zhang, 2015) and SWISS-MODEL server (Arnold et al., 2006; Waterhouse et al., 2018), using the crystal structure of the extracellular domain of CAR (a close relative of Clmp) as a template [PDB ID: 3JZ7; The Protein Data Bank (Berman et al., 2000)], which shows 32.84% sequence identity with mouse Clmp. Structural prediction of the C-terminal part of Clmp and IgSF11 proteins containing the last 10 amino acid sequences were further refined from the initial homology models using termini or loops modeling. Global optimization of an energy function composed of knowledge-based energy terms and physics-based energy terms was performed. The loop sampling efficiency was enhanced by generating proper closed-loop conformations with the triaxial loop closure (TLC) algorithm during global optimization (Ko et al., 2012; Park and Seok, 2012; Park et al., 2014). The surface electrostatic potential was calculated using PDB2PQR (v.2.1.1) (Dolinsky et al., 2004) and APBS (v.1.5) (Baker et al., 2001). All structural images were generated using PyMOL (The PyMOL Molecular Graphics System, Version 2.0 Schrödinger, LLC.) and UCSF Chimera (Pettersen et al., 2004).

### Generation of *Clmp*^–/–^ Mice

The *Clmp*^−/−^ mice used in the present study were originally generated by Genentech, Inc. (Tang et al., 2010). Briefly, to generate *Clmp* knockouts by homologous recombination, coding exons 3 through 5 targeted embryonic stem cells (Lexicon cell line derived from 129S5/SvEvBrd) were implanted into C57BL/6 albino-type blastocysts using standard procedures. The chimeric mice are bred to C57BL/6-Tyr^c−Brd^ albino mice albino mice to generate F1 heterozygous animals. These progenies were intercrossed to generate F2 wild type, heterozygous, and homozygous mutant progenies, which were further crossed for more than 6 generations. These *Clmp*^−/−^ mice were provided to us by the Mutant Mouse Resource & Research Centers (MMRRC:031613-UCD). We then backcrossed the heterozygous mice (N1; *Clmp*^+/−^) with C57BL/6J for at least 8 generations before being bred in the mixed background of C57BL/6J and 129S1/Sv (Jackson Labs) (50:50), as described previously (Zhou et al., 2016), to improve pre- and perinatal lethality. Littermates derived from heterozygous parents were used for all analysis. Genotyping of the *Clmp*^−/−^ mice was performed using PCR and the following four primers; P1 (5′- CGT ATT CCA GCC GTC ATG TC−3'′, P2 (5′- GGC TGT CTC TTG CCT CAT AG−3′), P3 (5′- TCC ACC GTA AAA GGA AGA CAA CC−3′), P4 (5′- GCA GCG CAT CGC CTT CTA TC−3′). The size of the PCR products for WT (P1 and P2) and mutant (*Clmp*^−/−^; P3 and P4) alleles were 514 and 209 bp, respectively. Both male and female mice were used for all measurements except behavioral experiments, which used only male mice. All mice were bred and maintained according to the KAIST Animal Research Requirements, and all procedures were approved by the Committees of Animal Research at KAIST (KA2016-32). Mice were fed *ad libitum* by standard rodent chow and tap water, and housed in specific pathogen-free condition under 12-h light/dark cycle (lights off at 19:00).

### Electrophysiology-Patch Recordings

Electrophysiological recordings for whole-cell patches were performed as previously described (Kim, 2009; Chung et al., 2015; Jang et al., 2016). Briefly, WT and *Clmp*^−/−^ mice at around week 1 (P7–11) were anesthetized by isoflurane inhalation. Acute sagittal dorsal hippocampal slices or acute horizontal middorsal hippocampal slices (300–400 μm thick for whole-cell recordings) were prepared using a vibratome (Leica VT1200s) in ice-cold high sucrose cutting solution containing (in mM) 212 sucrose, 25 NaHCO_3_, 2.5 or 5 KCl, 1.25 NaH_2_PO_4_, 0.5 CaCl_2_, 3.5 or 10 MgCl_2_, 10 D-glucose, 1.25 L-ascorbic acid, 2 Na-pyruvate equilibrated with 95% O_2_/5% CO_2_. Brain slices were then allowed to recover at 32°C for 30 min in artificial cerebral spinal fluid (aCSF) containing (in mM): 125 NaCl, 25 NaHCO_3_, 2.5 KCl, 1.25 NaH_2_PO_4_, 2.5 CaCl_2_, 1.3 MgCl_2_, 10 D-glucose) with pH 7.3–7.4 and osmolarity 296–300 mOsm and maintained at room temperature before recordings (0.5–1 h).

Whole-cell patch recordings were performed with recording pipettes pulled from borosilicate glass capillaries (Harvard Apparatus, 1.5 mm OD, GC150T*-*7.5) with a micropipette puller (Narishige PC-10). For whole-cell recordings, CA1 pyramidal cells, CA3 pyramidal cells, and DG granule cells were held at −70 mV with recording pipettes (4–5 MΩ) at 26°C (400 μm slice thickness) using Multiclamp 700B amplifier (Axon Instruments). For mEPSC recordings, Cs-based intracellular solution contained (in mM) 110 Cs-gluconate, 30 CsCl, 20 HEPES, 4 MgATP, 0.3 NaGTP, 4 NaVitC, 0.5 EGTA with pH 7.3 and osmolarity 295 mOsm. Picrotoxin (100 μM), D-AP5 (50 μM), and TTX (1 μM) were used to block inhibitory synaptic currents, N-methyl-D-aspartate receptor (NMDAR)-mediated synaptic currents and sodium channel-mediated action potentials, respectively. For consistent mEPSC measurements, the baseline was monitored for 5 min, and mEPSCs were began to be measured at the same time point (5–15 min) after whole-cell access was established to minimize time-dependent fluctuation. For DCG-IV CA3 mEPSCs, the group II mGluR agonist DCG-IV [(2S,2′R,3′R)-2-(2′,3′-Dicarboxycyclopropyl)glycine] (2 μM) was used in bath application. For LY354740 CA3 mEPSCs, the group II mGluR agonist LY354740 (0.5 μM) was used in bath application. For KAR mEPSCs, GYKI 53655 (30 μM) was used to block AMPAR receptor-mediated currents. Signals were filtered at 400 Hz to detect very small KAR mEPSCs ranging from 3 to 10 pA. For NMDAR mEPSCs, recordings were performed in 0 Mg^2+^ ACSF, the Mg^2+^ was replaced with additional glucose. NBQX (10 μM) was used to block AMPAR/KAR-mediated synaptic currents. Signals were filtered at 400 Hz to allow reliable detection of small NMDAR mEPSCs <10 pA. For mIPSC recordings, Cs-based intracellular solution contained (in mM) 120 CsCl, 10 TEA-Cl, 8 NaCl, 10 HEPES, 5 QX-314-Cl, 4 Mg-ATP, 0.3 Na-GTP, and 10 EGTA, with pH 7.35, 280 mOsm. TTX (1 μM), NBQX (10 μM), and D-AP5 (50 μM) was used to block spontaneous action potential-mediated synaptic currents, AMPAR/KAR-mediated synaptic currents and NMDAR-mediated synaptic currents, respectively. All miniature excitatory/inhibitory post-synaptic whole-cell recordings were performed in sagittal dorsal hippocampal slices. For mossy fiber (MF)-CA3 and recurrent associational/commissural (A/C)-CA3 EPSC recordings, acute horizontal middorsal hippocampal slices from WT and *Clmp*^−/−^ mice of at around week 1 (P9–11) were used to record mossy fiber-CA3 evoked synaptic responses (Marchal and Mulle, 2004; Bischofberger et al., 2006). The recording electrode was placed in the CA3 pyramidal cell layer but ~50 μm away from the stimulating electrode in the stratum lucidum of CA3. For pharmacological isolation of both AMPAR and KAR synaptic responses, picrotoxin (100 μM) and D-AP5 (50 μM) were used to block inhibitory synaptic currents and NMDAR-mediated synaptic currents, respectively. Cs-based intracellular solution containing (in mM) 110 Cs-gluconate, 30 CsCl, 20 HEPES, 4 MgATP, 0.3 NaGTP, 4 NaVitC, 0.5 EGTA with pH 7.3, and osmolarity 295 mOsm was used. For input-output experiments measuring MF-CA3 EPSCs and KAR EPSCs, input stimulus intensities ranged from 10 to 60 μA with 10 μA increments, and the output was the amplitude of the EPSCs averaged from five individual traces. AMPAR EPSCs were obtained by the subtraction of KAR EPSCs in the input-output experiments. To determine fast and slow decay kinetics of AMPAR- and KAR-mediated post-synaptic currents, respectively, the decay phase of MF-CA3 EPSCs was fitted to a double exponential function as previously described (Cossart et al., 2002; Pinheiro et al., 2013). Rise time values were assessed by measuring the time course from 20 to 80% of the total response amplitude. For slow rise kinetics of KAR-mediated post-synaptic currents, the fast rise kinetics of AMPAR-mediated post-synaptic currents were further pharmacologically blocked using GYKI 53655 (30 μM), a selective AMPAR antagonist (Paternain et al., 1995). For input-output experiments measuring NMDAR EPSCs, NBQX (10 μM) was used to block AMPA/KAR receptor-mediated currents and holding potential was changed to +40 mV to record NMDAR-mediated EPSCs. To determine NMDAR-mediated post-synaptic currents, the decay phase of NMDAR EPSCs was fitted to single exponential function. For extrasynaptic AMPAR experiments, AMPAR-mediated whole-cell currents were obtained by bath application of 1 μM (S)-α-amino-3-hydroxy-5-methyl-4-isoxazolepropionic acid (S-AMPA) for 5 min in the presence of 1 μM TTX and 100 μM picrotoxin after stable 5 min baseline holding currents were established. For extrasynaptic KAR experiments, KAR-mediated whole-cell currents were obtained by bath application of 3 μM kainate for 5 min in the presence of 1 μM TTX, 30 μM GYKI 53655, and 100 μM picrotoxin after stable 5 min baseline holding currents were established. For extrasynaptic NMDAR experiments, NMDAR-mediated whole-cell currents were obtained by bath application of 10 μM NMDA for 5 min in the presence of 1 μM TTX, 10 μM NBQX and 100 μM picrotoxin after stable 5 min baseline holding currents were established. All agonist-induced excitatory post-synaptic whole-cell recordings were performed in acute horizontal middorsal hippocampal slices. Liquid junction potentials were not corrected. Series access resistance was 10–25 MΩ, and only the cells with a change in series access resistance <25% were included in the analysis.

### Electrophysiology-Field Recordings

Electrophysiological recordings for extracellular field recordings were performed as previously described (Jang et al., 2016). Briefly, WT and *Clmp*^−/−^ mice at P 9–11 and weeks 8–16 were anesthetized by isoflurane inhalation. Acute horizontal middorsal hippocampal slices (400–500 μm thick) were used for extracellular field recordings at 28–30°C (TC-324B, Warner Instruments) (Bischofberger et al., 2006). Field excitatory post-synaptic potentials (fEPSPs) were recorded with glass electrodes (1–2 MΩ tip resistance) filled with ACSF or 1 M NaCl, and evoked every 20 s using a stimulating glass electrode filled with ACSF. For mossy fiber-CA3 fEPSP recordings, the recording electrode was placed in the stratum lucidum of CA3 but ~400 μm away from the stimulating electrode in the hilus of the DG region. To verify mossy fiber inputs, the strong paired-pulse facilitation of this input, a hallmark of MF-CA3 synapses, was measured by delivering paired pulses every 20 s. Paired pulse ratios were determined by evoking two fEPSPs (averages of three individual traces) that are 50 ms apart and dividing the initial slope of the second fEPSP by that of the first (fEPSP2/fEPSP1). For synaptic plasticity experiments, LTP at MF-CA3 synapses was induced by a single tetanus of 125 pulses at 25 Hz (Castillo et al., 1997a). At the end of the experiment, the degree of inhibition by the group II mGluR agonist DCG-IV [(2S,2′R,3′R)-2-(2′,3′-Dicarboxycyclopropyl)glycine] (2 μM) was used to verify that mossy fiber responses were recorded (Nicoll and Schmitz, 2005). For recurrent associational/commissural (A/C)-CA3 fEPSP recordings, the recording electrode was placed in the stratum radiatum of CA3 but 100–150 μm away from the stimulating electrode in the stratum radiatum of CA3 region. For perforant path (PP)-CA3 fEPSP recordings, the recording electrode was placed in the stratum lacunosum moleculare of CA3 but 100 μm away from the stimulating electrode in the stratum lacunosum moleculare of CA3 region.

### Electrophysiology-Data Acquisition and Analysis

All the recording data were digitized at 10 kHz and filtered at 2 kHz (except for the recording of KAR mEPSCs and NMDAR mEPSCs at 400 Hz). Analog-to-digital conversion was performed using Digidata 1440A (Molecular Devices). Data were acquired using Clampex 10 (Molecular Devices), and analyzed using Clampfit 10 (Axon Instruments) except that KAR mEPSCs were manually analyzed using MiniAnalysis Program 6.0.3 (Synaptosoft, Fort Lee, NJ, United States) to reliably detect the amplitude and the decaying phase of very small KAR-mediated miniature excitatory post-synaptic currents ranging from 3 to 10 pA. The experimenters were blind to the genotypes of the mice.

### Animal Behavioral Tests

All behavioral assays were performed using littermates or age-matched male animals during light-off periods. All behavioral tests were performed and analyzed in a blinded manner.

### Contextual Fear Conditioning Test

All experiments were carried out in a fear conditioning system (Coulbourn Instruments). Training and testing were performed in a chamber with a stainless steel grid floor. On the training day, mice at 2–6 months of age were placed in the fear chamber and allowed to freely move around the chamber for 2 min before they received five foot shocks as an unconditioned stimulus (US) (2 s, 0.7 mA, 1 min apart). The 10-s period before each US was used to measure inter-US freezing. 24 h later, animals were tested for contextual fear retrieval in context A for 5 min. Freezing behavior was quantified using an automatic detection system (FreezeFrame 3, Coulbourn Instruments). The animals were considered to freezing behavior if no movement was detected for 2 s, and freezing levels were indicated as the percentage of time spent freezing.

### Auditory-Cued Fear Conditioning and Contextual Fear Renewal Test

Mice at 2–6 months of age were submitted to an auditory fear conditioning paradigm. Two different contexts for conditioning (context A) and extinction (context B) were used. Context A and B were cleaned before and after each session with 70% ethanol or 1% acetic acid, respectively. During fear conditioning, the CS was paired to the US (2 s, 0.7 mA foot shock back to back with the last CS; 5 CS/US pairings; inter-trial interval: 60 s). Each behavior session consisted of a 10 min baseline prior to the presentation of the first conditioned stimulus (CS) (frequency: 8 kHz; sound pressure level: 75 dB), which gave the animal enough time to show normal locomotor activity after exposure to conditioning or extinction context. Two-min time prior to the first CS was used to measure baseline freezing. Each CS was 30-s long. CS inter-trial intervals were 60 s. After auditory fear conditioning in context A, animals were tested for cued retrieval in context B. Twenty-four hours later, mice were subjected to an extinction learning session composed of 20 identical tones (at intervals of 5 s) without shocks. Twenty-four hours later, animals were tested for extinction retrieval for 3 min in context B followed by contextual retrieval test for context-dependent fear renewal for 5 min in context A 1 h later.

### Pattern Completion-Based Contextual Fear Conditioning Test

Pre-exposure, pattern-completion version of contextual fear conditioning was previously described (Wagatsuma et al., 2018). Mice at 2–6 months of age were exposed to Context A for 10 min in the absence of shock on day 1. Immediate shock procedures were followed on day 2 in Context A, where a single 0.8 mA shock of 2 s duration was delivered at 8 s after being placed in the chamber, with all mice removed 30 s after the completion of the shock. On day 3, the mice were returned to the conditioning chamber for 5 min to recall contextual fear memory.

### Novel Object Recognition Test

Mice at 2–4 months of age were habituated in an open-field box without objects for 60 min a day before training session under low-light (15 lux) conditions. Object recognition test was performed in the same open-field box. On the first day, mice were allowed to explore two identical objects for 20 min. Twenty-four hours later, mice were placed the same box where one of the objects was replaced with a new object. Object recognition was scored manually by the amount of time with the nose of the mouse pointed and located within 2 cm from the object for 10 min.

### Kainate-Induced Seizure

Mice (2–6 months of age or post-natal day 10–12) were allowed to acclimate to a clean glass beaker containing bedding for 30 min under low-light (50 lux) conditions. After habituation to the testing room, mice received an intraperitoneal injection (20 mg/kg to adult male mice; 2 mg/kg to male and female neonatal mice) of kainic acid (Tocris 0222) in 1x phosphate-buffered saline (PBS; pH 7.4) (Vissel et al., 2001; Koh et al., 2004), and were monitored for 120 min following injection. Seizure levels were scored by an experimenter blinded to the genotype according to a modified Racine scale (Racine, 1972): stage 0, normal behavior; stage 1, behavioral arrest (absence-like immobility); stage 2, myoclonic seizure; stage 3, rearing with repetitive rhythmic bilateral forelimb clonus (2–3 Hz); stage 4, continuous rearing and falling; stage 5, generalized tonic-clonic seizure, jumping continues, and wild rushing; stage 6, death.

### Experimental Design

Experiments, data collection, and analyses were performed by researchers blinded to the experimental conditions. The following a priori criteria were established for appropriate exclusion of data points: cells that were morphologically unhealthy were excluded; in whole-cell experiments, cells that did not fulfill the standard criteria of electrophysiological properties, including cell capacitance, input resistance, series resistance, resting membrane potential, and baseline holding current, were excluded.

### Statistics

No statistical methods were used to predetermine sample sizes, but our sample sizes were similar to those generally employed in the field. All data were randomly collected. Normally distributed data were analyzed using the Student's *t*-test, whereas the data that did not conform to a normal distribution were analyzed using the non-parametric Mann-Whitney test. Outliers were determined using ROUT test and removed from the analysis. Statistical tests and data point plotting were performed using GraphPad Prism 7. All details of statistical analyses are described in Supplementary Table 1.

## Results

### Widespread Expression of Clmp mRNAs in the Mouse Brain

**Figure 1:**
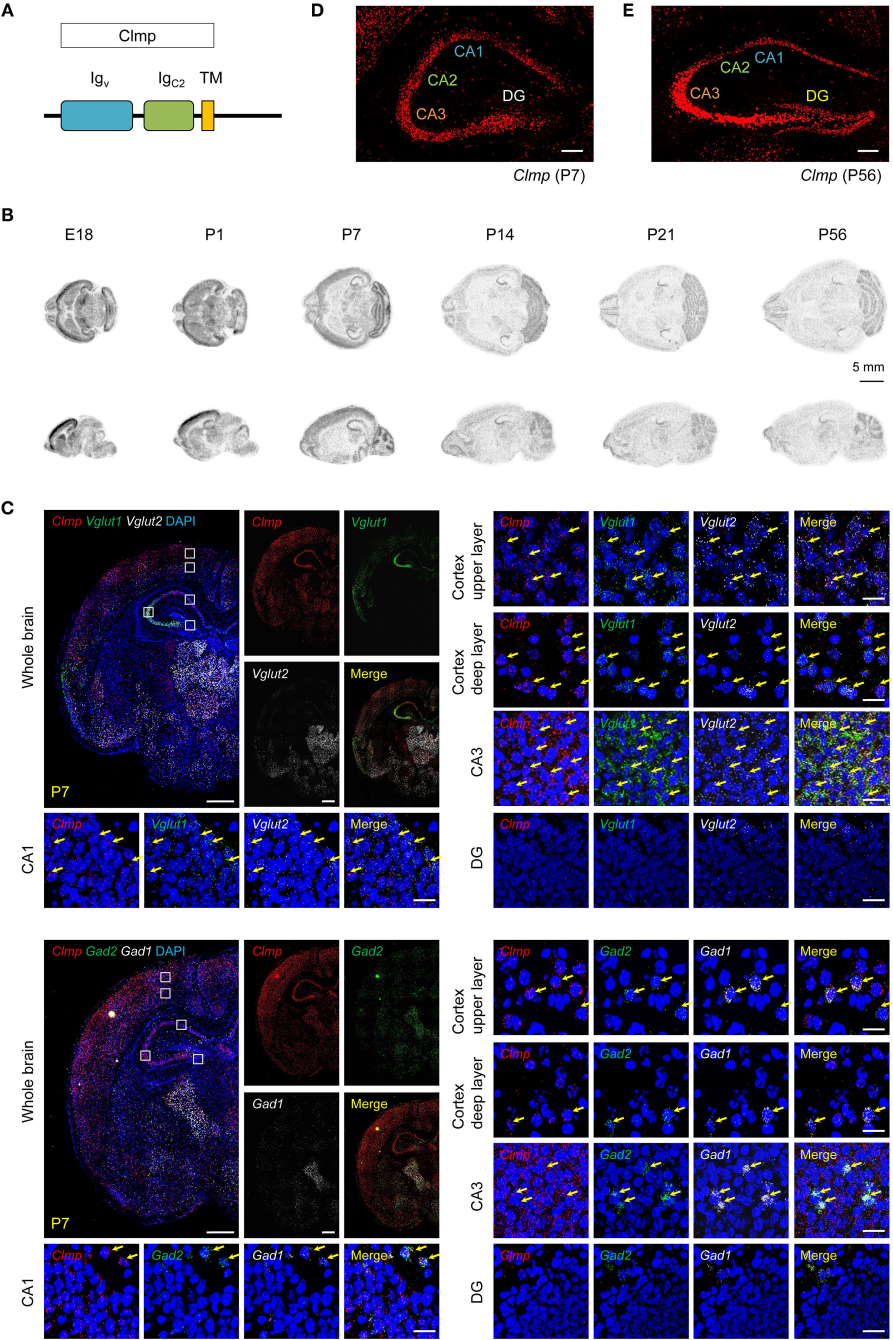
Distribution patterns of Clmp mRNAs. **(A)** Domain structure of Clmp. Ig_v/C2_, immunoglobulin V/C2-like; TM, transmembrane. **(B)** Distribution patterns of Clmp mRNA in the mouse brain at E18 and P1, P7, P14, P21, and P56, as revealed by isotopic *in situ* hybridization. **(C)** Expression of Clmp mRNA in Vglut1/2-positive glutamatergic neurons (upper panels) and Gad1/2-positive GABAergic neurons (lower panels) in the neocortex and hippocampus in the mouse brain [week 1 (P7)], as revealed by fluorescence *in situ* hybridization (FISH). Coronal brain sections were doubly stained for Clmp and Vglut1/2 or Gad1/2, and counterstained with DAPI (nuclear stain; blue). Images at right show enlarged views of white boxes in the images at left. The yellow arrows indicate neurons that express both Clmp and neuronal markers. Note that the DG area shows faint signals for Clmp mRNA. Scale bar, 300 μm (left) and 20 μm (right). **(D,E)** Age-dependent changes in the distribution patterns of Clmp mRNA in the hippocampus of the mouse brain at weeks 1 and 8, revealed by FISH. Note that the DG area shows strong Clmp mRNA signals at week 8, but not at week 1. Scale bar, 200 μm.

Clmp, a member of the immunoglobulin superfamily, is a type I transmembrane protein that is conserved among various vertebrate species (Supplementary Figure 1). Clmp, which is ~373 amino acid (aa)-long in humans and mice, contains an extracellular region with two immunoglobulin-like (Ig) domains (one V-type and one C2-type), a single transmembrane domain, and an intracellular region (Figure 1A).

To determine the expression patterns of the *Clmp* gene in the mouse brain, we first characterized the distribution patterns of Clmp mRNA in the mouse brain by *in situ* hybridization. Radioisotope probes revealed a widespread distribution pattern of Clmp mRNA in the mouse brain at various developmental stages [embryonic day 18 and post-natal (P) days 1, 7, 14, 21, and 56], including the olfactory bulb, cortex, striatum, hippocampus, thalamus, and cerebellum (Figure 1B). A separate fluorescence *in situ* hybridization (FISH) analyses revealed a similar widespread distribution of Clmp mRNA in mouse brain regions, including the cortex, hippocampus and thalamus, at two different post-natal stages: week 1 (P7) and week 8 (P56) (Figure 1C; Supplementary Figure 2).

Notably, Clmp mRNA was abundant in the CA1, CA2, and CA3 regions of the hippocampus, but not in dentate gyrus (DG) regions, at week 1 (Figure 1D). In contrast, at week 8, Clmp mRNA was also detected in the DG area, in addition to other hippocampal regions (Figure 1E). This indicates an age-dependent change in Clmp expression in the hippocampal DG area.

We also tested whether Clmp mRNA is differentially expressed in glutamatergic and GABAergic neurons using the FISH technique. These experiments revealed the presence of Clmp mRNA in both types of neurons in brain regions that included the cortex and hippocampus at two different post-natal stages (week 1 and 8) (Figure 1C; Supplementary Figure 2).

### Clmp Protein Is Enriched in Synaptic, but Not PSD, Fractions, and Fails to Interact With PSD-95

**Figure 2:**
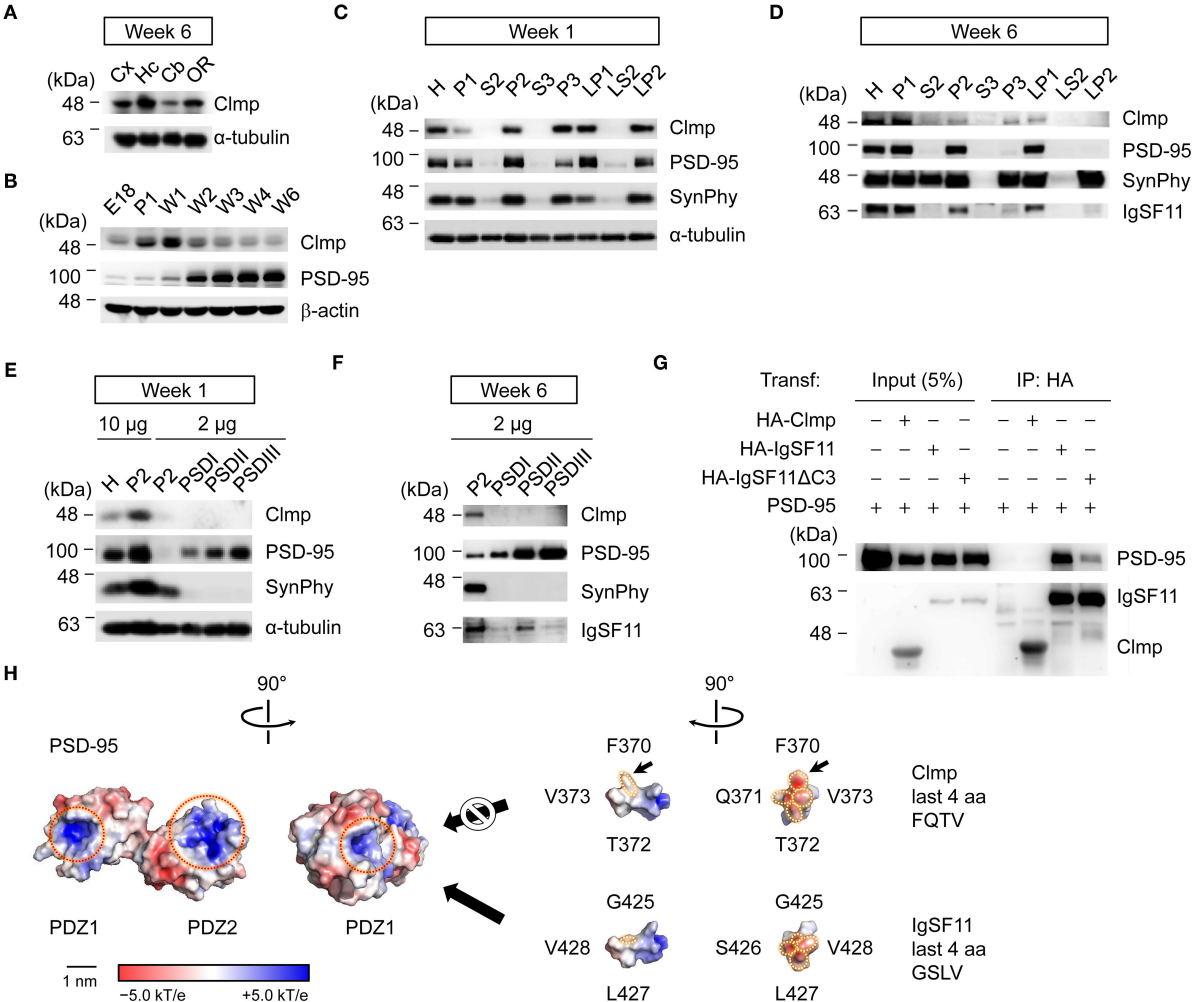
Clmp protein is enriched in synaptic, but not PSD, fractions and fails to interact with PSD-95. **(A)** Levels of Clmp proteins in different brain regions (6 weeks, rat), revealed by immunoblot analysis. Cx, cortex; Hc, hippocampus; Cb, cerebellum; OR, other brain regions. **(B)** Changes in Clmp protein levels during stages of post-natal brain developmental (rat), revealed by immunoblot analysis. PSD-95, a control post-synaptic protein; β-actin, a loading control. **(C,D)** Subcellular fractionation patterns of Clmp protein in the rat brain (weeks 1 and 6), revealed by immunoblot analysis. Note that Clmp protein is more abundant in synaptic membrane and vesicle fractions (P2, P3, LP1, and LP2) at week 1 and also at week 6, although at this latter stage, LP2 levels are strongly reduced. H, homogenates; P1, cells and nuclei-enriched pellet; P2, crude synaptosomes; S2, supernatant after P2 precipitation; S3, cytosol; P3, light membranes; LP1, synaptosomal membranes; LS2, synaptosomal cytosol; LP2, synaptic vesicle-enriched fraction. **(E,F)** Minimal enrichment of Clmp proteins in PSD fractions in the rat brain (weeks 1 and 6). PSD I, Triton X-100 once; PSD II, Triton X-100 twice; PSD III, Triton X-100 and sarcosyl. **(G)** Clmp does not form a complex with PSD-95 in HEK293T cells. IgSF11, a positive control for PSD-95 association; IgSF11 ΔC3, a mutant IgSF11 that lacks PSD-95 binding; Transf, Transfection; IP, immunoprecipitation; HA, HA-tag. **(H)** Molecular modeling indicates steric hindrance between the F residue in the Clmp C-terminus and the tail-binding pocket in the PDZ1 domain of PSD-95, predicted using the structures of the PDZ1 and PDZ2 domains of PSD-95 (PDB 3GSL) and the last 10 aa residues of Clmp and IgSF11. Dotted red circles, tail-binding pockets in the PDZ domain; dotted white circles, last four aa residues of Clmp and IgSF11. The surface is color-coded based on electrostatic potential, contoured from −5.0 kT/e (red: acidic) to +5.0 kT/e (blue: basic). Hydrophobic surface patches are shown in white. Scale bar, 1 nm.

Clmp protein (~48 kDa) was detected at relatively high levels in the hippocampus compared with other brain regions (Figure 2A). Levels of Clmp protein rapidly increased after birth, reaching a peak at approximately week 1 and decreasing to adult levels around week 3, an expression pattern that contrasted with that of PSD-95, a major excitatory post-synaptic protein (Sheng and Sala, 2001), which steadily increased until reaching a peak at about week 3 (Figure 2B).

Biochemical fractionation experiments on whole mouse brain samples at week 1 indicated that Clmp protein is enriched in synaptic fractions, including crude synaptosomes (P2), the synaptic vesicle fraction (P3), the synaptosomal membrane fraction (LP1), and the synaptosomal vesicle fraction (LP2) (Figure 2C). Subcellular distribution patterns at week 6 were similar to those at week 1, although levels of Clmp protein in the LP2 fraction were decreased at week 6 (Figure 2D). Intriguingly, Clmp protein was not enriched in post-synaptic density (PSD) fractions at week 1 or 6 (Figures 2E,F). These results contrast sharply with the abovementioned enrichment of Clmp protein in synaptic membrane and synaptic vesicle fractions, suggesting that Clmp protein is present at synaptic sites but is not tightly associated with the PSD.

IgSF11, a relative of Clmp, binds to the PDZ domains (PDZ1 + PDZ2) of PSD-95 through its C-terminal PDZ-binding motif (Jang et al., 2016). The C-terminal tail of the Clmp protein containing the last four residues, FQTV, partly resembles the Class I PDZ domain-binding motif (X-S/T-X-V) (Sheng and Sala, 2001). We thus tested whether Clmp could directly bind to and form a complex with PSD-95 in heterologous cells. Clmp failed to bind PSD-95, whereas IgSF11 did form a complex with PSD-95 in a manner that required the last four amino acid (aa) residues (Figure 2G). Molecular modeling indicated steric hindrance between the F residue in the FQTV sequence of the Clmp tail and the tail-binding pocket of the PDZ1 domain of PSD-95 (Figure 2H), which is predicted to suppress the interaction. These results indicate that Clmp is unable to bind PSD-95, a major component of the PSD, in line with the limited enrichment of Clmp in PSD fractions.

### Increased AMPAR and KAR mEPSCs and Decreased NMDAR mEPSCs in Clmp^-/-^ Hippocampal CA3 Neurons

Clmp protein is detected at synaptic sites, despite not being enriched in PSD fractions, and previous studies indicate that the Clmp relatives, IgSF11, and CAR, regulate excitatory synaptic transmission in the hippocampus (Jang et al., 2016; Wrackmeyer et al., 2019). We thus tested if *Clmp* deletion in mice alters synaptic transmission in the hippocampus, where Clmp is most strongly expressed (Figure 2A). To this end, we generated mice carrying a homozygous deletion of exons 3–5 of the *Clmp* gene (*Clmp*^−/−^) (Supplementary Figures 3A,B). Complete loss of Clmp protein was confirmed by immunoblot analyses using whole-brain lysates of the *Clmp*^−/−^ brain and an antibody directed against the last 29 aa residues of the protein generated as part of the present study (Supplementary Figure 3C). At P7, *Clmp*^−/−^ mice showed a Mendelian ratio of 0.30:0.50:0.20 (WT, *Clmp*^+/−^, and *Clmp*^−/−^), indicative of a reduction in the proportion of *Clmp*^−/−^ mice compared with the expected ratio of 0.25:0.50:0.25 and similar to a previous report (Langhorst et al., 2018). In line with this, *Clmp*^−/−^ mice displayed decreased (~50%) survival rate during post-natal stages (Supplementary Figure 3D).

**Figure 3:**
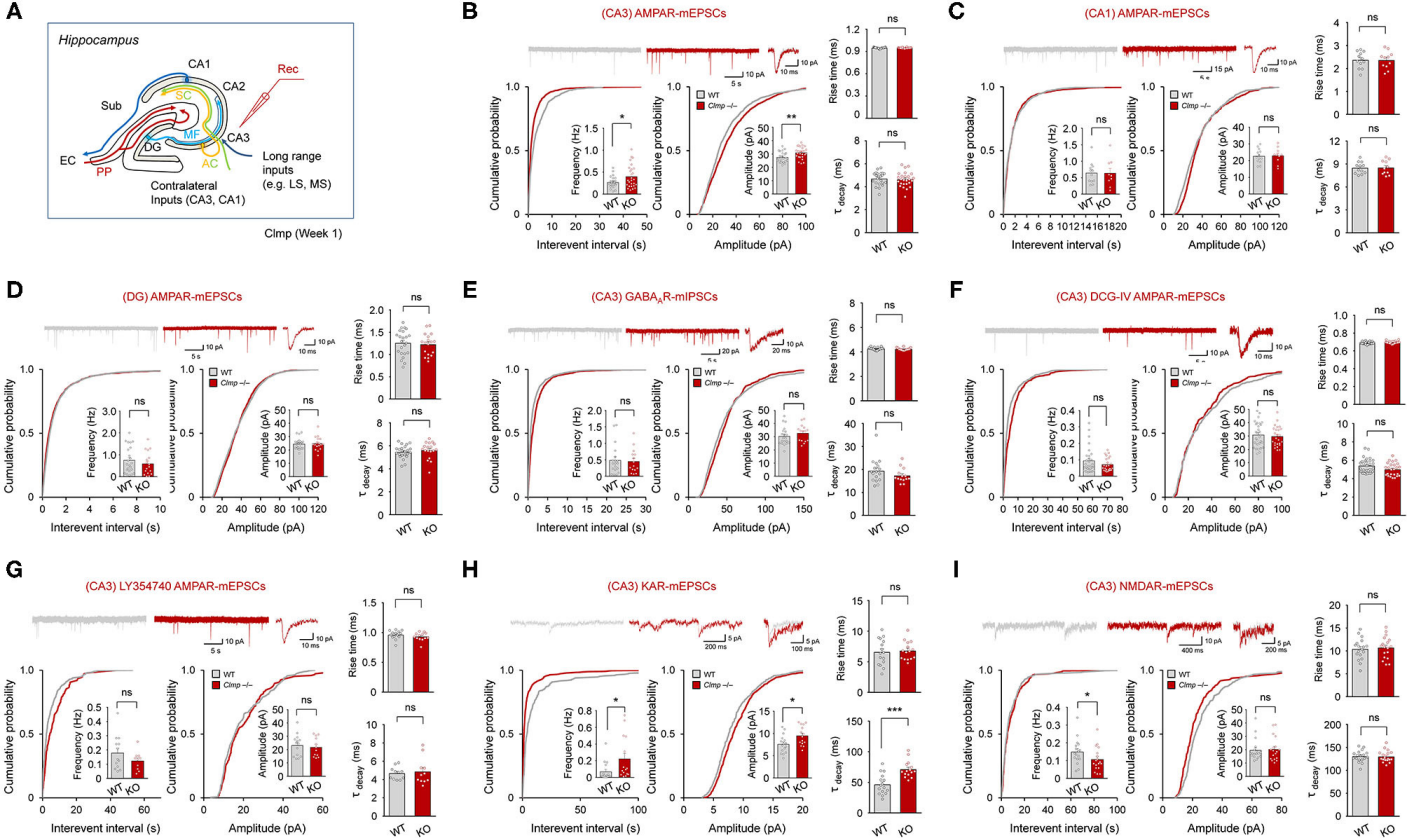
Increased AMPAR and KAR mEPSCs and decreased NMDAR mEPSCs in *Clmp*^−/−^ hippocampal CA3 neurons. **(A)** Schematic diagram showing synaptic pathways in the hippocampus. PP, performant pathways; MF, mossy fiber pathway; AC, associational commissural pathway; SC, Schaffer collateral pathway; LS/MS, lateral/medial septum; EC, entorhinal cortex; DG, dentate gyrus; CA1/2/3, Cornu Ammonis area 1/2/3; Sub, subiculum. **(B)** Increased frequency and amplitude but unaltered rise time and decay time constant in AMPAR mEPSCs in CA3 pyramidal neurons in *Clmp*^−/−^ mice (week 1). Data are presented as means ± SEM. [*n* = 23 neurons from 7 mice (WT) and 26, 7 (KO), *P < 0.05, **P < 0.01, Student's *t*-test (frequency and amplitude)]. **(C)** Normal AMPAR mEPSC frequency, amplitude, rise time, and decay time constant in CA1 pyramidal neurons in *Clmp*^−/−^ mice (week 1). [*n* = 13, 4 mice (WT), and 10, 4 (KO), ns, not significant, Student's *t*-test (frequency and amplitude)]. **(D)** Normal AMPAR mEPSC frequency, amplitude, rise time, and decay time constant in DG cells in *Clmp*^−/−^ mice (week 1). [*n* = 23, 5 mice (WT), and 17, 5 (KO), ns, not significant, Student's *t*-test (frequency and amplitude)]. **(E)** Normal AMPAR mIPSC frequency, amplitude, rise time, and decay time constant in CA3 pyramidal neurons in *Clmp*^−/−^ mice (week 1). [*n* = 20, 4 (WT), and 15, 4 (KO), ns, not significant, Student's *t*-test (frequency and amplitude)]. **(F)** No genotype difference between WT and *Clmp*^−/−^ CA3 pyramidal neurons (week 1) in the frequency and amplitude of AMPAR mEPSCs measured in the presence of with DCG-IV treatment (2 μM) throughout recordings. [*n* = 30, 8 (WT), and 25, 8 (KO), ns, not significant, Student's *t*-test (frequency and amplitude)]. **(G)** No genotype difference between WT and *Clmp*^−/−^ CA3 pyramidal neurons (week 1) in the frequency and amplitude of AMPAR mEPSCs measured in the presence of with LY354740 treatment (0.5 μM) throughout recordings. [*n* = 14, 4 (WT), and 13, 4 (KO), ns, not significant, Student's *t*-test]. **(H)** Increases in the frequency, amplitude, and decay time constant, but unaltered rise time, in KAR mEPSCs in CA3 pyramidal neurons in *Clmp*^−/−^ mice (week 1). Data are presented as means ± SEM. [*n* = 16 neurons from 8 mice (WT) and 15, 8 (KO), *P < 0.05, ***P < 0.001, Student's *t*-test]. **(I)** Decreased frequency but normal amplitude, rise time, and decay time constant in NMDAR mEPSCs in CA3 pyramidal neurons in *Clmp*^−/−^ mice (week 1). Data are presented as means ± SEM. [*n* = 18 neurons from 4 mice (WT) and 18, 4 (KO), *P < 0.05, Student's *t*-test].

We next examined miniature post-synaptic currents in *Clmp*^−/−^ hippocampal regions, including CA1, CA3 and the DG, at week 1 (Figure 3A), the developmental stage at which *Clmp* expression peaks. *Clmp*^−/−^ pyramidal neurons in the CA3 region showed increases in both the frequency and amplitude of miniature excitatory post-synaptic currents (mEPSCs) (Figure 3B). In contrast, *Clmp*^−/−^ pyramidal neurons in the CA1 and DG region showed unaltered mEPSCs (Figures 3C,D). Inhibitory synaptic transmission, determined by measuring miniature inhibitory post-synaptic currents (mIPSCs), was unaltered in CA3 neurons (Figure 3E), indicative of a selective increase in excitatory transmission. Importantly, DCG-IV, a selective agonist of group II metabotropic glutamate receptors (mGluR2/3) that is known to reduce synaptic transmission at mossy fiber inputs but not at associational inputs in the CA3 region (Yoshino et al., 1996), fully reversed the increased mEPSC frequency and amplitude in *Clmp*^−/−^ CA3 pyramidal neurons (Figure 3F), by more strongly decreasing mEPSCs in *Clmp*^−/−^ than in WT CA3 neurons (82% vs. 65% in mEPSC frequency) (Figures 3B,F). In addition, another mGluR2/3 agonist, LY354740, eliminated the genotype differences in the frequency and amplitude of mEPSCs between WT and *Clmp*^−/−^ CA3 neurons (Figure 3G), by less strongly decreasing *Clmp*^−/−^ mEPSCs (69 vs. 34% in mEPSC frequency) (Figures 3B,G).

The abovementioned mEPSCs in different hippocampal regions, including CA3, are mainly mediated by AMPARs. We thus tried to isolate mEPSCs mediated by KARs or NMDARs. KAR-mEPSCs, isolated by the selective AMPAR antagonist GYKI 53655 and the NMDAR blocker AP5, showed increases in the frequency and amplitude as well as in the decay time constant but not in the rise time (Figure 3H). In addition, NMDAR-mEPSCs, isolated by the AMPAR/KAR antagonist NBQX, revealed a decrease in the frequency but not amplitude (Figure 3I). Together, these results collectively suggest that *Clmp* deletion leads to changes in AMPAR, KAR, and NMDAR mEPSCs in *Clmp*^−/−^ CA3 neurons; increased frequency and amplitude of AMPAR mEPSCs, increased frequency, amplitude, and decay time constant of KAR mEPSCs, and decreased frequency of NMDAR mEPSCs.

### Moderately Changed Kinetics of Evoked Excitatory Synaptic Transmission at Clmp^-/-^ MF-CA3 Synapses

**Figure 4:**
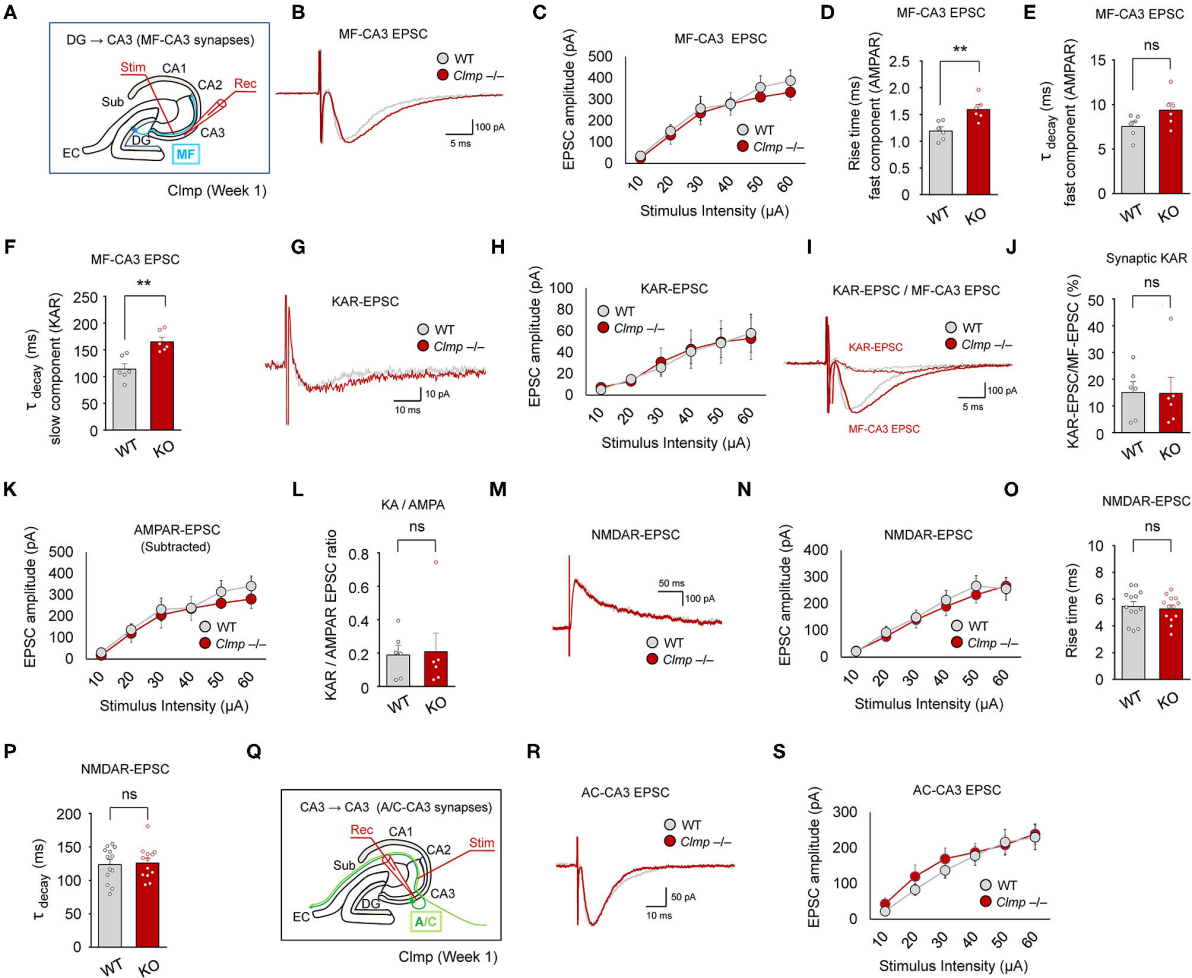
Moderately changed kinetics of evoked excitatory synaptic transmission at *Clmp*^−/−^ MF-CA3 synapses. **(A)** Schematic diagram showing the MF-CA3 pathway and sites of electrical stimulation and evoked EPSC (eEPSC) measurements. **(B,C)** Normal amounts of eEPSCs at MF-CA3 synapses of *Clmp*^−/−^ mice (week 1), as indicated by the input-output relationship between eEPSC amplitudes plotted against stimulus intensities. Data are presented as means ± SEM. [*n* = 6 neurons from 6 mice (WT and KO), ns, not significant, two-way ANOVA with Sidak's multiple comparison test]. **(D–F)** Increased fast-rise time constant (involving AMPARs), unaltered fast-decay time constant (involving AMPARs), and increased slow-decay time constant (involving KARs) of eEPSCs at MF-CA3 synapses of *Clmp*^−/−^ mice (week 1). [*n* = 6, 6 (WT and KO), ns, not significant, **P < 0.01, ns, not significant, Student's *t*-test]. **(G,H)** Normal amounts of KAR eEPSCs at MF-CA3 synapses of *Clmp*^−/−^ mice (week 1), as indicated by the input-output relationship between KAR eEPSC amplitudes plotted against stimulus intensities. [*n* = 6, 6 (WT and KO), ns, not significant, two-way ANOVA with Sidak's test]. **(I,J)** Normal ratios of KAR eEPSCs and total eEPSCs at MF-CA3 synapses of *Clmp*^−/−^ mice (week 1). [*n* = 6, 6 (WT and KO), ns, not significant, Student's *t*-test]. **(K)** Normal amounts of AMPAR eEPSCs, obtained by subtracting KAR eEPSCs from total eEPSCs, at MF-CA3 synapses of *Clmp*^−/−^ mice (week 1), as indicated by the input-output relationship between AMPAR eEPSC amplitudes plotted against stimulus intensities. [*n* = 6, 6 (WT and KO), ns, not significant, two-way ANOVA with Sidak's test]. **(L)** Normal ratios of KAR- and AMPAR-mediated eEPSCs (KA/AMPA ratio) at MF-CA3 synapses of *Clmp*^−/−^ mice (week 1). [*n* = 6, 6 (WT and KO), ns, not significant, Student's *t*-test]. **(M,N)** Normal amounts of NMDAR eEPSCs at MF-CA3 synapses of *Clmp*^−/−^ mice (week 1). [*n* = 13, 6 (WT and KO), ns, not significant, two-way ANOVA with Sidak's multiple comparison test]. **(O,P)** Normal rise and decay time constants in NMDAR eEPSCs at MF-CA3 synapses of *Clmp*^−/−^ mice (week 1). [*n* = 13, 6 (WT and KO), ns, not significant, Student's *t*-test]. **(Q–S)** Normal amounts of eEPSCs at A/C-CA3 synapses of *Clmp*^−/−^ mice (week 1), as indicated by the input-output relationship between eEPSC amplitudes plotted against stimulus intensities. [*n* = 16, 14 (WT), and 15, 13 (KO), ns, not significant, two-way ANOVA with Sidak's multiple comparison test].

Because the abovementioned results suggest that Clmp negatively regulates miniature excitatory post-synaptic currents at CA3 synapses, we tested whether Clmp also negatively regulates evoked excitatory transmission in MF-CA3 pathway, one of the principal inputs to the CA3 neurons. The amount of evoked excitatory post-synaptic current (eEPSCs), measured in the presence of the NMDAR antagonist AP5, was normal at *Clmp*^−/−^ MF-CA3 synapses, as indicated by the input-output curve of EPSC amplitudes plotted against stimulus intensities (Figures 4A,B). Notably, however, *Clmp*^−/−^ eEPSCs displayed increased fast-rise and slow-decay (but not fast-decay) time constants (Figures 4C–F) characteristic of the kinetic properties of AMPAR- and KAR-mediated eEPSCs, respectively.

Isolation of KAR-mediated evoked EPSCs using the AMPAR antagonist GYKI 53655, which were sensitive to NBQX (AMPAR and KAR antagonist) (Supplementary Figure 4), revealed that the peak amplitude of KAR eEPSCs was unaltered at *Clmp*^−/−^ MF-CA3 synapses (Figures 4G,H); kinetics of KAR eEPSCs were not analyzed because of the apparent presence of multiple components. Consistent with this, the ratio of KAR eEPSCs to total eEPSCs was unaltered at *Clmp*^−/−^ MF-CA3 synapses (Figures 4I,J). In addition, AMPAR eEPSCs, obtained by subtracting KAR eEPSCs from total eEPSCs, and the ratio of KAR to AMPAR eEPSCs were unchanged at MF-CA3 synapses (Figures 4K,L). NMDAR-mediated EPSCs at *Clmp*^−/−^ MF-CA3 synapses, isolated by NBQX treatment, showed unaltered peak amplitude and kinetic parameters (rise time and decay constant) (Figures 4M–P).

In the A/C-CA3 pathway, AMPAR/KAR eEPSCs were unaltered in *Clmp*^−/−^ mice (Figures 4Q–S). AMPAR/KAR eEPSCs in the PP-CA3 pathway were not analyzed because of the small sizes of eEPSCs. These results collectively suggest that *Clmp* deletion induces a moderate change in the kinetics of AMPAR/KAR eEPSCs without affecting the peak amplitudes of AMPAR, KAR, or NMDAR eEPSCs at MF-CA3 synapses.

### Extracellular and Cytoplasmic Regions of Clmp Interact With AMPARs and KARs

**Figure 5:**
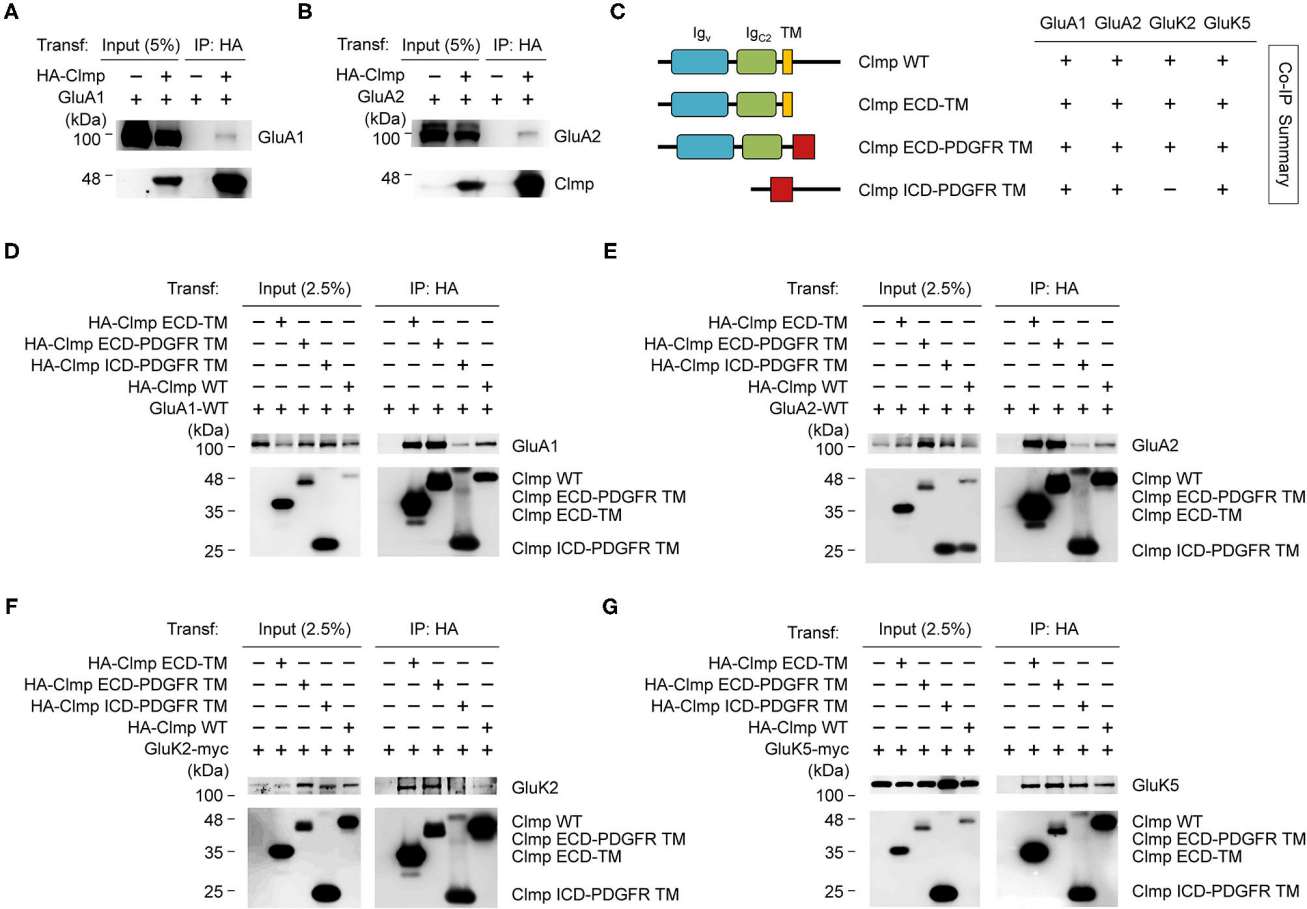
Extracellular and cytoplasmic regions of Clmp interact with AMPARs and KARs. **(A,B)** Clmp forms a complex with GluA1 and GluA2 subunits of AMPARs. HEK293T cells transfected with HA-Clmp and GluA1/2, or GluA1/2 alone, were subjected to immunoprecipitation (IP) with HA antibodies and immunoblotting with the indicated antibodies. HA-IgSF11, N-terminally HA-tagged Clmp. **(C)** Schematic diagram of Clmp deletion variants, and summary of coimmunoprecipitation experiments using these variants to test binding to GluA and GluK. ECD-TM, extracellular domains of Clmp + transmembrane domain of Clmp; ECD-PDGFR TM, extracellular domains of Clmp + transmembrane domain from unrelated PDGF receptor; ICD-PDGFR TM, intracellular/cytoplasmic domain of Clmp + transmembrane domain from unrelated PDGF receptor. **(D,E)** All Clmp deletion variants form a complex with AMPAR subunits (GluA1 and GluA2). HEK293T cells transfected with the HA-tagged deletion variants of Clmp and GluA1/2, or GluA1/2 alone, were subjected to immunoprecipitation with HA antibodies and immunoblotting. **(F,G)** All Clmp deletion variants form a complex with GluK2 and GluK5, with the exception of Clmp-ICD (cytoplasmic domain), which showed no binding to GluK2. HEK293T cells transfected with the HA-tagged deletion variants of Clmp and GluK2/5, or GluK2/5 alone, were subjected to immunoprecipitation with HA antibodies and immunoblotting.

How does Clmp affect the kinetics of AMPAR- and KAR-mediated EPSCs? One possibility involves the interaction of Clmp with AMPARs and KARs. To address this possibility, we tested whether Clmp formed a complex with AMPAR or KAR subunits in heterologous cells. Notably, we found that Clmp indeed formed a complex with GluA1 and GluA2 subunits of AMPARs in HEK293T cells (Figures 5A,B).

We next used deletion variants of Clmp to identify the domains involved in AMPAR/KAR interactions (Figure 5C). Deletion mutants of Clmp containing only the extracellular Ig domains, Ig domains + transmembrane domain, or cytoplasmic domain were able to associate with GluA1 or GluA2, although the extracellular Ig domains seemed to contribute more strongly (Figures 5C–E). These results suggest that both extracellular and cytoplasmic regions of Clmp contribute to GluA1/2 binding.

In the case of KAR subunits, all deletion variants of Clmp could associate with GluK5, similar to the results from GluA1/2, although GluK2 failed to associate with a Clmp variant containing only the cytoplasmic domain (Figures 5F,G). These results suggest that the extracellular domains of Clmp are more important than the cytoplasmic domain for interacting with GluK2, but not GluK5. Together, these results suggest that, with the exception of GluK2, Clmp associates with AMPARs and KARs through both extracellular and cytoplasmic domains.

### A Clmp Deficiency Does Not Alter Presynaptic Function or Synaptic Plasticity at MF-CA3 Synapses

**Figure 6:**
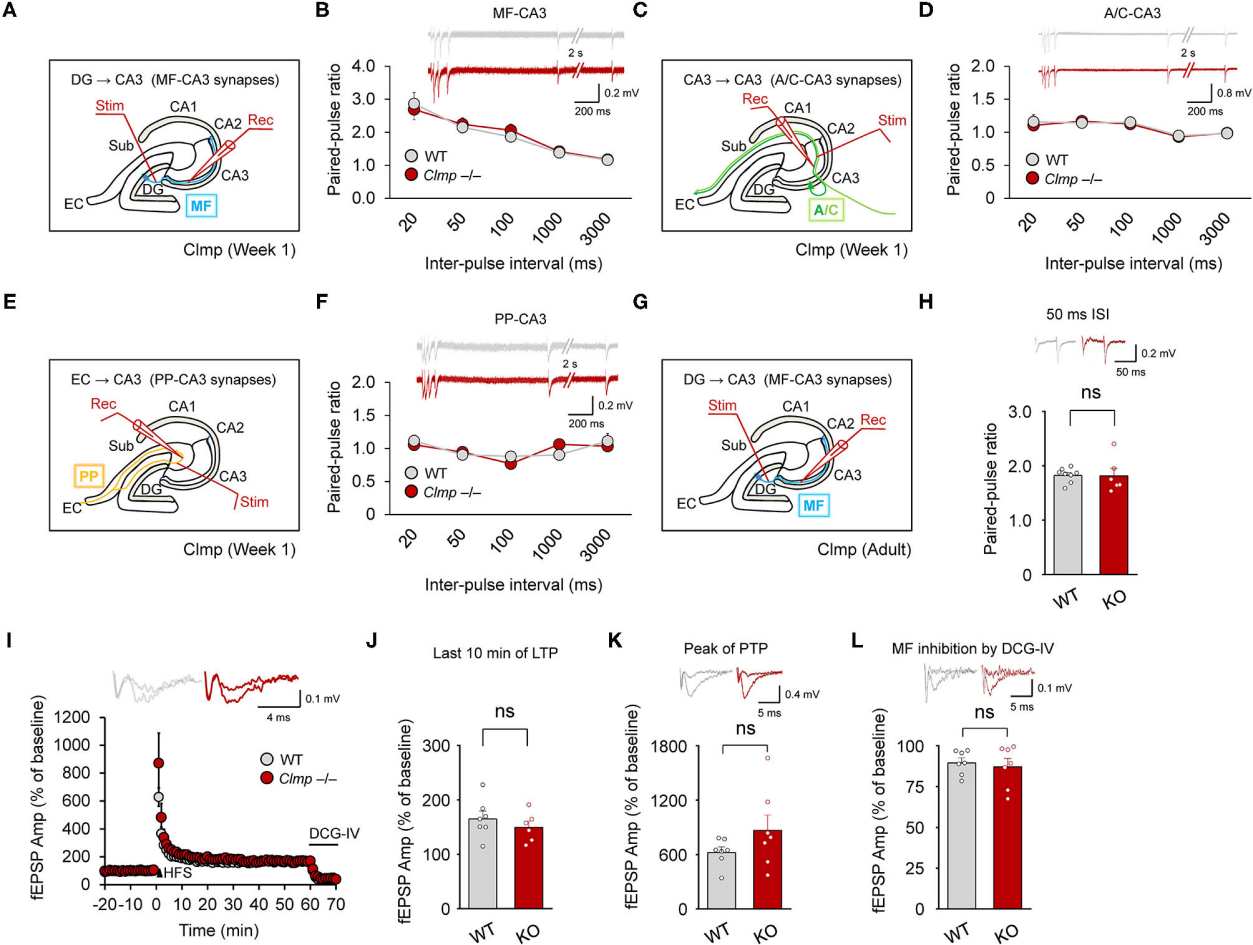
Normal presynaptic function and synaptic plasticity at *Clmp*^−/−^ CA3 synapses. **(A,B)** Normal paired-pulse ratios at MF-CA3 synapses of *Clmp*^−/−^ mice (week 1). [*n* = 7 slices from 7 mice (WT and KO), ns, not significant, two-way ANOVA with Sidak's multiple comparison test]. **(C,D)** Normal paired-pulse ratios at AC (associational commissural)-CA3 synapses of *Clmp*^−/−^ mice (week 1). [*n* = 7, 7 (WT and KO), ns, not significant, two-way ANOVA with Sidak's multiple comparison test]. **(E,F)** Normal paired-pulse ratios at PP (performant path)-CA3 synapses of *Clmp*^−/−^ mice (week 1). [*n* = 6, 6 (WT and KO), ns, not significant, two-way ANOVA with Sidak's multiple comparison test]. **(G–L)** Normal LTP and PTP at MF-CA3 synapses of *Clmp*^−/−^ mice (8–16 weeks). LTP was induced by a single train of high-frequency stimuli (125 pulses at 25 Hz). Synaptic transmission at MF-CA3 synapses was validated by inhibition with the group II mGluR agonist DCG-IV **(I,L)** and by the observed high paired-pulse ratios **(H)**. [*n* = 7, 7 (WT), and 7, 6 (KO), ns, not significant, Student's *t*-test (last 10 min)].

We next examined whether *Clmp* deletion affects presynaptic function at MF-CA3 synapses, associational commissural (AC)-CA3 synapses, or perforant path (PP)-CA3 synapses. We found that paired-pulse ratios were not changed in any of these CA3 synapse types in the *Clmp*^−/−^ hippocampus at week 1 (Figures 6A–F).

KARs represent one of the major glutamate receptor subtypes that have been implicated in the regulation of long-term potentiation (LTP) at MF-CA3 synapses (Nicoll and Schmitz, 2005), and our data indicate changes in the kinetic properties of KARs at these synapses in *Clmp*^−/−^ mice (Figure 3). Thus, we asked whether Clmp affects synaptic plasticity at MF-CA3 synapses (Figure 6G). To this end, we measured MF-CA3 LTP at weeks 8–16, a time chosen because Clmp mRNA is detectable in presynaptic neurons (DG granule cells) of the MF-CA3 pathway at week 8 but not at week 1, and LTP at MF-CA3 synapses mainly involves presynaptic mechanisms (Rebola et al., 2017).

However, we detected no genotype difference in LTP at MF-CA3 synapses (Figures 6H,I) or post-tetanic potentiation (PTP) (Figure 6J) of excitatory synaptic transmission at MF-CA3 synapses, as validated by the inhibition of synaptic transmission by the group II mGluR agonist DCG-IV (Figure 6K) and high paired-pulse ratios (Figure 6L). Together, these results suggest that Clmp does not affect presynaptic function or synaptic plasticity at MF-CA3 synapses.

### Increased Extrasynaptic KAR Currents and Decreased NMDAR Currents in *Clmp^−/−^* Hippocampal CA3 Neurons

The abovementioned alterations in AMPAR- and KAR-mediated synaptic currents may involve changes in extrasynaptic pools of these receptors. To this end, we measured extrasynaptic AMPAR-, KAR- and NMDAR-mediated currents in *Clmp*^−/−^ CA3 neurons by applying AMPA, kainate, or NMDA to CA3 neurons in slices and measuring the ligand-induced currents (mainly extrasynaptic).

**Figure 7:**
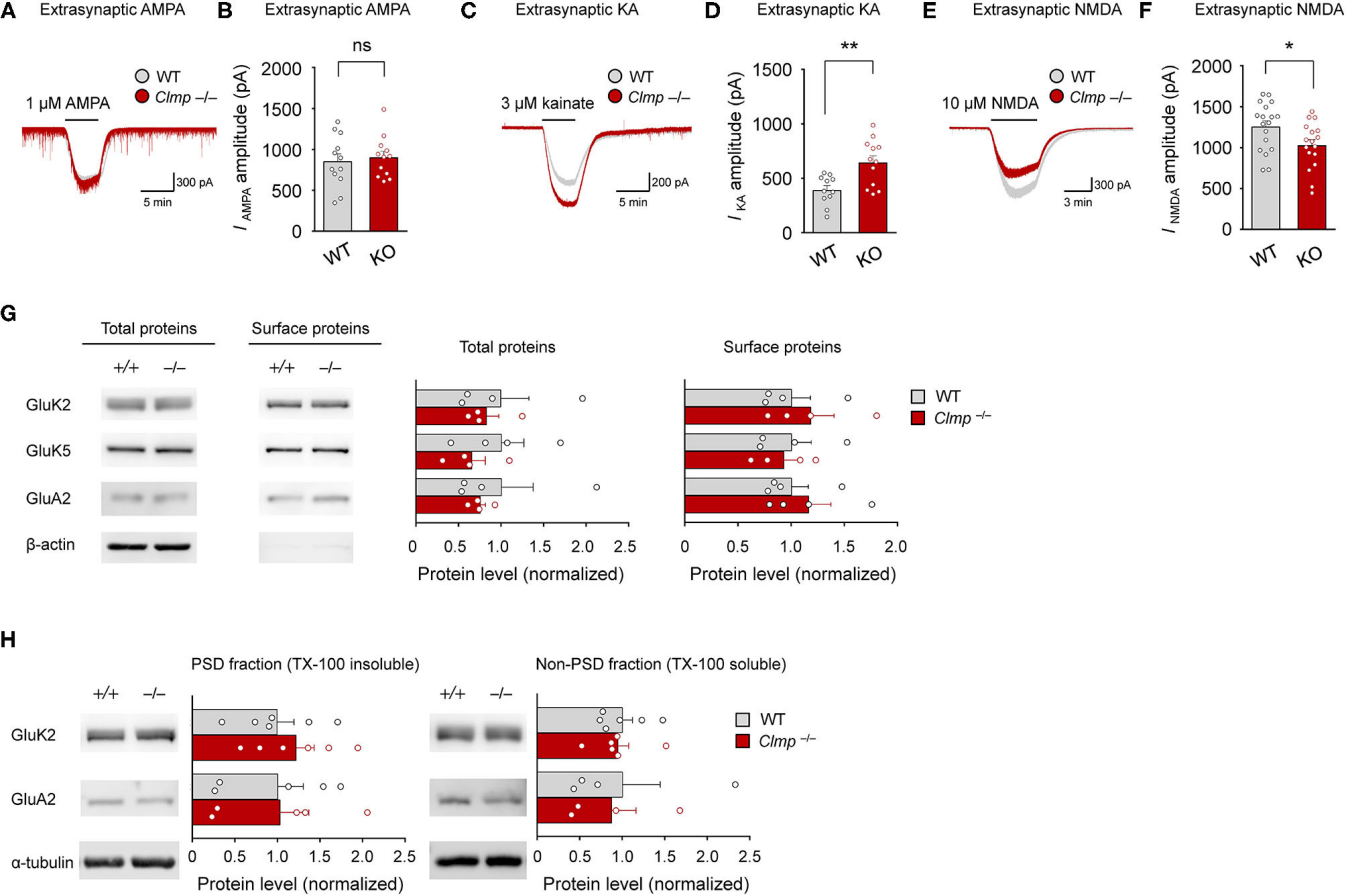
Increased extrasynaptic KAR currents and decreased extrasynaptic NMDAR currents in *Clmp*^−/−^ hippocampal CA3 neurons. **(A,B)** Normal extrasynaptic AMPAR currents in *Clmp*^−/−^ CA3 neurons (1 week), as determined peak currents induced AMPA treatment. [*n* = 12 neurons from 12 mice (WT and KO), ns, not significant, Student's *t*-test]. **(C,D)** Increased extrasynaptic KAR currents in *Clmp*^−/−^ CA3 neurons (1 week), as determined peak currents induced kainate treatment. [*n* = 11 neurons from 9 mice (WT) and 12, 8 (KO), **P < 0.01, Student's *t*-test]. **(E,F)** Decreased extrasynaptic NMDAR currents in *Clmp*^−/−^ CA3 neurons (1 week), as determined peak currents induced NMDA treatment. [*n* = 18 neurons from 3 mice (WT) and 17, 3 (KO), *P < 0.05, Student's *t*-test]. **(G)** Comparable levels of total and surface AMPAR and KAR receptor subunit proteins in the *Clmp*^−/−^ hippocampus (P10–11), as determined by immunoblot analyses of total lysates and avidin/biotin precipitates of surface proteins from the hippocampus. Note that the minimal levels of β-catenin in the surface samples support the authenticity of the samples. [*n* = 4 mice (WT and KO), ns, not significant, Student's *t*-test]. **(H)** Comparable levels of PSD-enriched AMPAR and KAR receptor subunit proteins in *Clmp*^−/−^ whole brains (P10–11), as determined by immunoblot analyses of PSD fractions. [*n* = 6 mice (WT and KO) except for GluA2 (*n* = 5 for PSD and 4 for non-PSD), ns, not significant, Student's *t*-test].

Intriguingly, while AMPAR currents were unaltered, KAR currents were increased, and NMDAR currents were decreased (Figures 7A–F). These changes, however, did not accompany detectable changes in the levels of AMPAR or KAR subunits at the surface membrane of the hippocampus or in PSD fractions of whole brains (Figures 7G,H). These results suggest that Clmp deficiency alters the extrasynaptic functions of AMPARs and KARs in CA3 pyramidal neurons without affecting the surface or synaptic expression levels of these receptors.

### Enhanced Susceptibility to Kainate-Induced Seizures and Increased Novel Object-Recognition Memory

Clmp protein is abundant in the hippocampus (Figure 2A), and *Clmp* deletion alters miniature and evoked excitatory post-synaptic currents in the hippocampal CA3 region (Figures 3, 4). We thus tested whether *Clmp* deletion affects hippocampus-dependent learning and memory or AMPA and kainate receptor-dependent seizure responses in mice.

**Figure 8:**
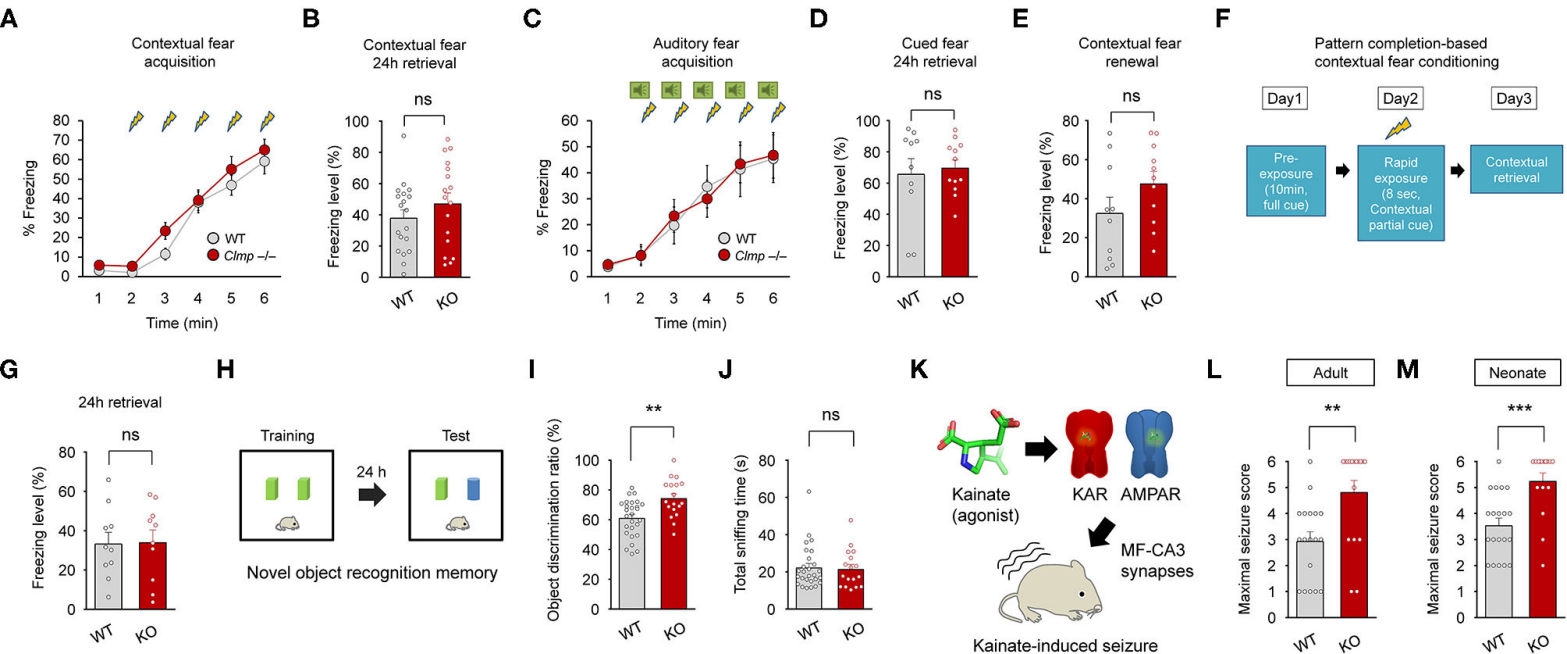
Enhanced object recognition memory and increased susceptibility to kainate-induced seizures in *Clmp*^−/−^ mice. **(A,B)** Normal contextual fear memory acquisition and 24-h retrieval in *Clmp*^−/−^ mice (2–6 months). [*n* = 19 mice (WT) and 17 (KO), ns, not significant, two-way ANOVA with Sidak's test (acquisition) and Student's *t*-test (24-h retrieval)]. **(C–E)** Normal cued fear memory acquisition, 24-h retrieval, and contextual fear renewal in *Clmp*^−/−^ mice (2–4 months). [*n* = 10 (WT) and 12 (KO), ns, not significant, two-way ANOVA with Sidak's test (acquisition) and Student's *t*-test (24-h retrieval and renewal)]. **(F,G)** Normal pattern completion-based contextual fear conditioning in *Clmp*^−/−^ mice (2–4 months). A subject mouse fully exposed to context A on day 1 was briefly (8 s) exposed to the same context on day 2, followed by measurements of context- and pattern completion-dependent freezing on day 3. [*n* = 10 (WT and KO), ns, not significant, Student's *t*-test]. **(H–J)** Normal novel object recognition memory in *Clmp*^−/−^ mice (2–4 months), as shown by the discrimination ratio for old and new objects. Total sniffing time was determined as a measure of general motivation for object exploration. [*n* = 28 (WT) and 18 (KO), **P < 0.01, ns, not significant, Student's *t*-test]. **(K–M)** Increased susceptibility to kainate-induced seizures in adult and neonatal *Clmp*^−/−^ mice (2–6 months and P10–12). [*n* = 18 (WT) and 17 (KO) for 2–6 months, 20 (WT), and 16 (KO) for P10–12, **P < 0.01, ***P < 0.001, Student's *t*-test].

*Clmp*^−/−^ mice showed normal levels of contextual fear memory acquisition and 24-h retrieval (Figures 8A,B). In the auditory-cued fear test, *Clmp*^−/−^ mice showed normal fear memory acquisition, 24-h retrieval, and contextual fear renewal after auditory-cued fear memory extinction (Figures 8C–E). *Clmp*^−/−^ mice also showed normal levels of pattern completion-based contextual fear memory (Figures 8F,G), which is known to require intact CA3 function (Nakazawa et al., 2004; Neunuebel and Knierim, 2014).

Intriguingly, however, *Clmp*^−/−^ mice performed better in the novel object-recognition test, in which the subject mouse is exposed to two identical objects on day 1, followed by replacement of one of the two objects with a new one on day 2 (Figures 8H,I). The total object-sniffing time for *Clmp*^−/−^ mice was normal, indicating unaltered motivation for object exploration (Figure 8J). These results show that *Clmp* deletion does not affect contextual or auditory cued fear memory or pattern completion-based contextual fear memory, but does improve object-recognition memory.

Lastly, because *Clmp* deletion leads to increased miniature excitatory post-synaptic currents in CA3 pyramidal cells and alters the kinetics of AMPAR- and KAR-mediated evoked excitatory synaptic transmission, we tested whether *Clmp*^−/−^ mice display altered susceptibility to seizures induced by kainate (a KAR agonist and a partial AMPAR agonist at MF-CA3 synapses), modeling temporal lobe epilepsy (Nadler, 1981; Engel, 1996; Ben-Ari and Cossart, 2000). Notably, *Clmp*^−/−^ mice at both adult and neonatal stages displayed increased susceptibility to kainate-induced seizure (Figures 8K–M). Together, these results suggest that *Clmp* deletion leads to enhanced object recognition memory and increased susceptibility to kainate-induced seizures in mice.

## Discussion

The present study investigated the spatiotemporal expression patterns of Clmp, protein interactions of Clmp, and Clmp-dependent regulation of synaptic transmission and behaviors. Our results reveal several properties of Clmp that are unique relative to other members of the CAR subgroup of Ig superfamily proteins (IgSF11, CAR, and ESAM). First, Clmp expression reached a peak at around post-natal week 1 (Figure 2B), whereas IgSF11 expression steadily increases during post-natal brain development (Jang et al., 2016), and CAR expression reaches a peak around birth (Honda et al., 2000). Second, Clmp does not have a functional PDZ-binding motif, as supported by protein-interaction experiments and molecular modeling (Figures 2G,H), whereas IgSF11, CAR, and ESAM have canonical PDZ-binding motifs that interact with PDZ domain-containing proteins. Third, Clmp regulates synaptic transmission in the hippocampal CA3 region, whereas IgSF11 and CAR regulate synaptic transmission in DG (Jang et al., 2016) and CA1 (Wrackmeyer et al., 2019) regions, respectively. Fourth, Clmp interacts with AMPAR subunits (GluA1 and GluA2) and KAR subunits (GluK2 and GluK5) (Figure 5), whereas IgSF11 interacts with AMPAR subunits (GluA1 and GluA2) but not with NMDAR subunits (GluN1); IgSF11 was not tested for KAR subunit interactions (Jang et al., 2016). CAR was not tested for the interactions with AMPAR, KAR, or NMDAR subunits (Wrackmeyer et al., 2019). These results indicate that CAR subgroup members have distinct spatiotemporal expression patterns, synapse-regulatory functions, and molecular interactions with ionotropic glutamate receptors.

Our results indicate that *Clmp* deletion leads to altered AMPAR responses in *Clmp*^−/−^ CA3 neurons. The increased frequency and amplitude of AMPAR mEPSCs (Figure 3B) could be explained by increased excitatory synapse number/maturation or increased presynaptic release. The latter, however, is an unlikely possibility because paired pulse facilitations at three different pathways (MF-CA3, A/C-CA3, and PP-CA3) onto CA3 neurons were not altered in *Clmp*^−/−^ mice (Figures 5A–H). Therefore, the former (enhanced excitatory synapse development) is the likely possibility. These changes at the individual synapse level, however, does not seem to be reflected at evoked synaptic AMPAR/KAR currents measured at *Clmp*^−/−^ MF-CA3 or A/C-CA3 synapses (Figures 4A–C,Q–S) or extrasynaptic AMPAR currents in *Clmp*^−/−^ CA3 neurons (Figures 7A,B). It is possible that other synaptic pathways onto CA3 pyramidal neurons such as PP-CA3 may be enhanced, although evoked PP-CA3 currents were not analyzed for their small sizes. Notably, eEPSCs at *Clmp*^−/−^ MF-CA3 synapses showed increased rise time (Figure 4D), suggesting that Clmp might regulate the kinetics of AMPAR eEPSCs, although AMPAR mEPSCs did not show altered kinetic properties (Figure 3B). These results collectively suggest that Clmp may negatively regulate the development of AMPAR-containing excitatory synapses in CA3 neurons.

*Clmp* deletion also leads to altered KAR responses in *Clmp*^−/−^ CA3 neurons. Similar to the changes in AMPAR mEPSCs, *Clmp*^−/−^ KAR mEPSCs show increases in the frequency, amplitude, and decay time constant (Figure 3H). Peak amplitudes of KAR eEPSCs were also unchanged in the *Clmp*^−/−^ MF-CA3 pathway (Figures 4G,H), again similar to the results of AMPAR eEPSC measurements. Distinctly, however, extrasynaptic KAR currents were strongly increased in *Clmp*^−/−^ CA3 neurons, contrary to the unaltered extrasynaptic AMPAR currents (Figures 7A–D), suggesting the possibility that Clmp may suppress the response properties of KARs such as kainate binding. Therefore, Clmp seems to similarly suppress the development of AMPAR- and KAR-containing excitatory synapses but distinctly suppress response properties of extrasynaptic KARs not AMPARs. Whether these changes would involve the interactions of Clmp with AMPARs/KARs, which employee slightly different domains of Clmp (Figure 5), remains to be determined.

*Clmp* deletion does not affect LTP at MF-CA3 synapses in the *Clmp*^−/−^ hippocampus (Figures 6I,J). This is line with the lack of changes in hippocampus-dependent memory, including contextual fear memory, auditory cued fear memory, contextual fear renewal, and pattern completion-based contextual fear conditioning (Figures 8A–G). Intriguingly, however, *Clmp*^−/−^ mice showed increased novel-object recognition (Figures 8H–J). Increasing evidence suggests that the dorsal hippocampus is important for novel object recognition memory (Broadbent et al., 2010; Antunes and Biala, 2012; Patten et al., 2015; Liu et al., 2016), although one study reported that the hippocampal CA3 region is less important for object recognition memory in mice (Stupien et al., 2003). Notably, a recent study has reported a novel object-dependent increase in c-fos activity in the DG hilar region (Bernstein et al., 2019), a brain region where Clmp is expressed in the adult, but not neonatal, stage (Figure 1E; Supplementary Figures 2A,B). However, previous studies have associated novel-object recognition memory with brain regions other than the DG, including the lateral entorhinal cortex, perirhinal cortex, and hippocampal CA1 region (Barker and Warburton, 2011; Wilson et al., 2013; Warburton and Brown, 2015; Furini et al., 2020). Therefore, the brain regions accounting for the enhanced novel object recognition in *Clmp*^−/−^ mice might involve both hippocampal and non-hippocampal brain regions expressing Clmp. In addition, the synaptic changes induced by Clmp deletion in neonatal CA3 neurons may represent compensatory changes and thus not be causally linked to the enhanced novel object recognition observed in adult *Clmp*^−/−^ mice.

*Clmp*^−/−^ mice also displayed increased susceptibility to kainate-induced seizures in both neonates and adults (Figures 8K–M). Our electrophysiological results are mainly obtained from neonates but not from adults because peak expression of *Clmp* was observed at ~P7. However, the similar kainate-induced seizures in *Clmp*^−/−^ neonates and adults suggest that the CA3 region may also be excitable in *Clmp*^−/−^ adults. The hippocampal CA3 region has been closely linked to susceptibility to kainate-induced seizures. For instance, mice lacking the NMDAR subunit GluN1 in the CA3 region show increased susceptibility to kainate-induced seizures (Fukushima et al., 2009; Jinde et al., 2009). Because synaptic transmission in CA3 pyramidal cells is required for kainate-induced seizure activity (Yu et al., 2016), the enhanced synaptic transmission in the *Clmp*^−/−^ CA3 region may contribute to the increased susceptibility to kainate-induced seizures. Notably, Clmp has not been linked to epilepsy. However, the *CLMP* gene is expressed in the developing human cerebral neocortex in regions including the hippocampus, striatum, amygdala, thalamus, and cerebellum (Kang et al., 2011; Pletikos et al., 2014). In addition, synaptic adhesion molecules have been increasingly associated with epilepsy (Gorlewicz and Kaczmarek, 2018), suggesting the possible association of CLMP with epilepsy as well.

In conclusion, our study identifies Clmp as a novel synaptic cell adhesion molecule involved in the negative regulation of AMPAR/KAR-mediated synaptic transmission in the CA3 region of the neonatal hippocampus. In addition, our results implicate Clmp in the regulation of object recognition and seizure susceptibility. Further studies on Clmp will help elucidate the mechanisms underlying synaptic and AMPAR/KAR regulation and behavioral and brain abnormalities.

## Data Availability Statement

The original contributions presented in the study are included in the article/Supplementary Materials, further inquiries can be directed to the corresponding author/s.

## Ethics Statement

The animal study was reviewed and approved by all animals were bred and maintained according to the Requirements of Animal Research at KAIST, and all procedures were approved by the Committees of Animal Research at KAIST (KA2016-32).

## Author Contributions

SJ performed the biochemical experiments, structural prediction analyses, electrophysiological experiments, mouse breeding and behavioral experiments, and generated *Clmp*^−/−^ mice. SJ and DK performed designed constructs. DK made *in situ* probe. EY performed *in situ* hybridization experiments. SJ and EK designed the experiments and wrote the manuscript. All authors contributed to the article and approved the submitted version.

## Conflict of Interest

The authors declare that the research was conducted in the absence of any commercial or financial relationships that could be construed as a potential conflict of interest.

## Supplementary Material

Supplementary Figure 1 Amino acid sequence alignment of Clmp proteins from various vertebrate species. **(A)** Amino acid residues in black and gray backgrounds indicate fully and partially conserved residues, respectively. The following aa sequences were used for comparison; human (NP_079045.1), chimpanzee (JAA42653.1), macaque monkey (XP_001108111.1), marmoset (JAB43212.1), rat (NP_775177.1), mouse (NP_598494.2), finch (ENSTGUT00000022861.1), turtle (XP_006116688.1), lizard (ENSACAT00000004856.3), and zebrafish (XP_695564.4). TM, transmembrane domain.

Supplementary Figure 2 Expression of Clmp mRNA in both glutamatergic and GABAergic neurons in the mouse brain at week 8. **(A,B)** Expression of Clmp mRNA in Vglut1/2-positive glutamatergic neurons **(A)** and Gad1/2-positive GABAergic neurons **(B)** in the neocortex and hippocampus in the mouse brain [week 8 (P56)], as detected by FISH. Coronal brain sections were triply stained for Clmp, Vglut1/2 or Gad1/2, and DAPI. Note that the DG area show strong signals for *Clmp* mRNAs. Scale bar, 300 μm (left) and 20 μm (right).

Supplementary Figure 3 Generation and characterization of *Clmp*^−/−^ mice. **(A)** Schematic diagram showing the strategy for generating *Clmp*^−/−^ mice lacking exons 3–5 of the *Clmp* gene. P1, P2, P3, and P4, primers for PCR genotyping. **(B)** Genotyping of wild-type (*Clmp*^+/+^; WT), *Clmp*^+/−^, and *Clmp*^−/−^ mice by PCR. **(C)** *Clmp*^−/−^ mice show no detectable levels of Clmp proteins in the whole brain (week 8). β-actin blot was used as a control. **(D)** Survival curve of *Clmp*^−/−^ mice during post-natal stages [*n* = 28 mice (+/+; WT), 76 (+/–; hetero), and 31 (**-**/**-**; KO)].

Supplementary Figure 4 Elimination of KAR eEPSCs by NBQX at MF-CA3 synapses. **(A)** KAR eEPSCs isolated by the treatment of the slices with GYKI 53655 (AMPAR antagonist), AP5 (NMDAR antagonist), and picrotoxin (GABA receptor antagonist) were eliminated by the treatment of NBQX (AMPAR and KAR antagonist), as shown by two examples of KAR eEPSCs recorded at WT MF-CA3 synapses (week 1).

Supplementary Table 1 Statistical Details.
