# Supplementary figures and images for: Clmp Regulates AMPA and Kainate Receptor Responses in the Neonatal Hippocampal CA3 and Kainate Seizure Susceptibility in Mice
Source: Front Synaptic Neurosci. 2020 Dec 21;12:567075. doi: 10.3389/fnsyn.2020.567075 (PMC7779639; doi:10.3389/fnsyn.2020.567075)

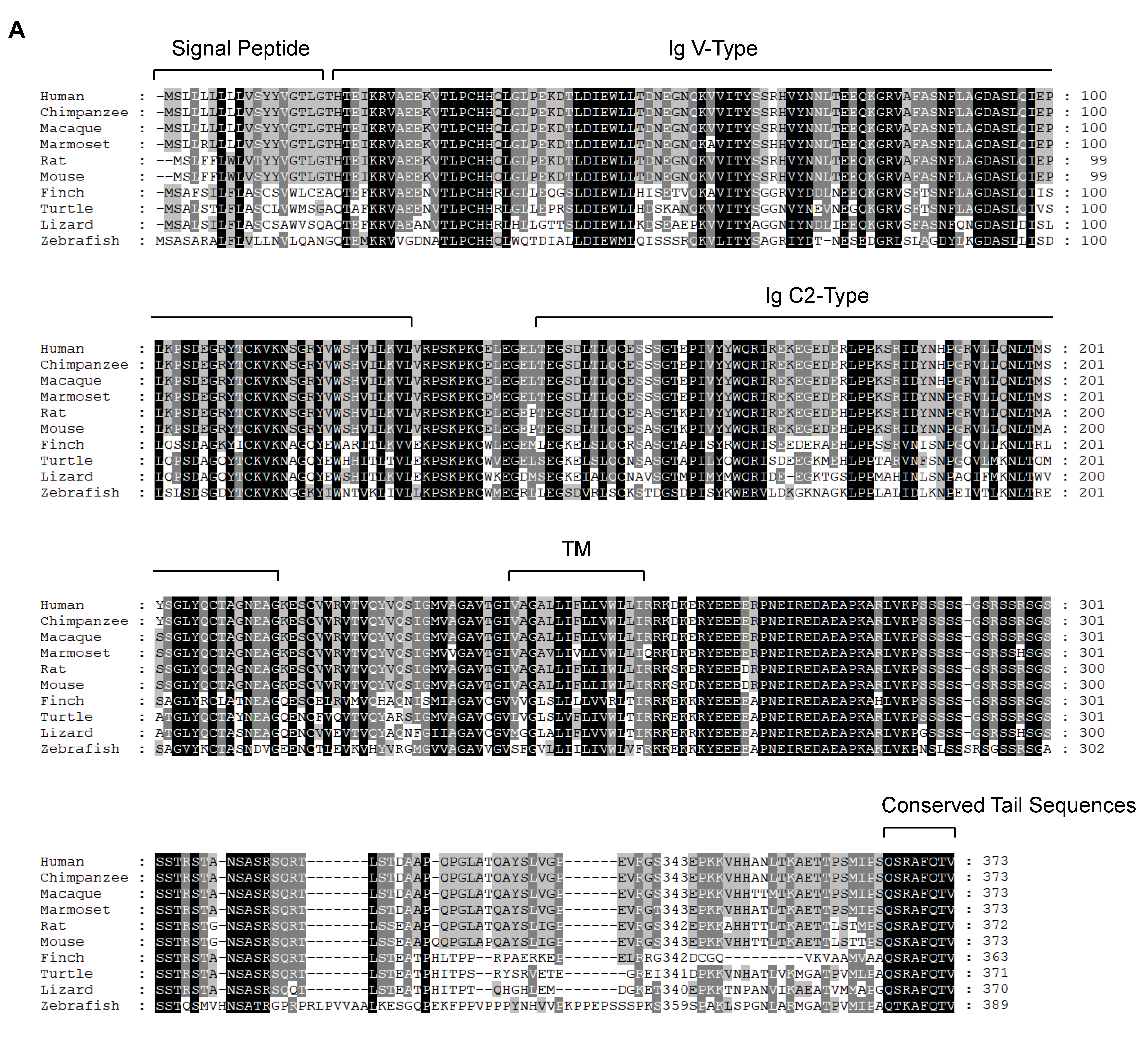

Supplement: Supplementary Figure 1 — Amino acid sequence alignment of Clmp proteins from various vertebrate species. (A) Amino acid residues in black and gray backgrounds indicate fully and partially conserved residues, respectively. The following aa sequences were used for comparison; human (NP_079045.1), chimpanzee (JAA42653.1), macaque monkey (XP_001108111.1), marmoset (JAB43212.1), rat (NP_775177.1), mouse (NP_598494.2), finch (ENSTGUT00000022861.1), turtle (XP_006116688.1), lizard (ENSACAT00000004856.3), and zebrafish (XP_695564.4). TM, transmembrane domain. [file Image_1.JPEG]

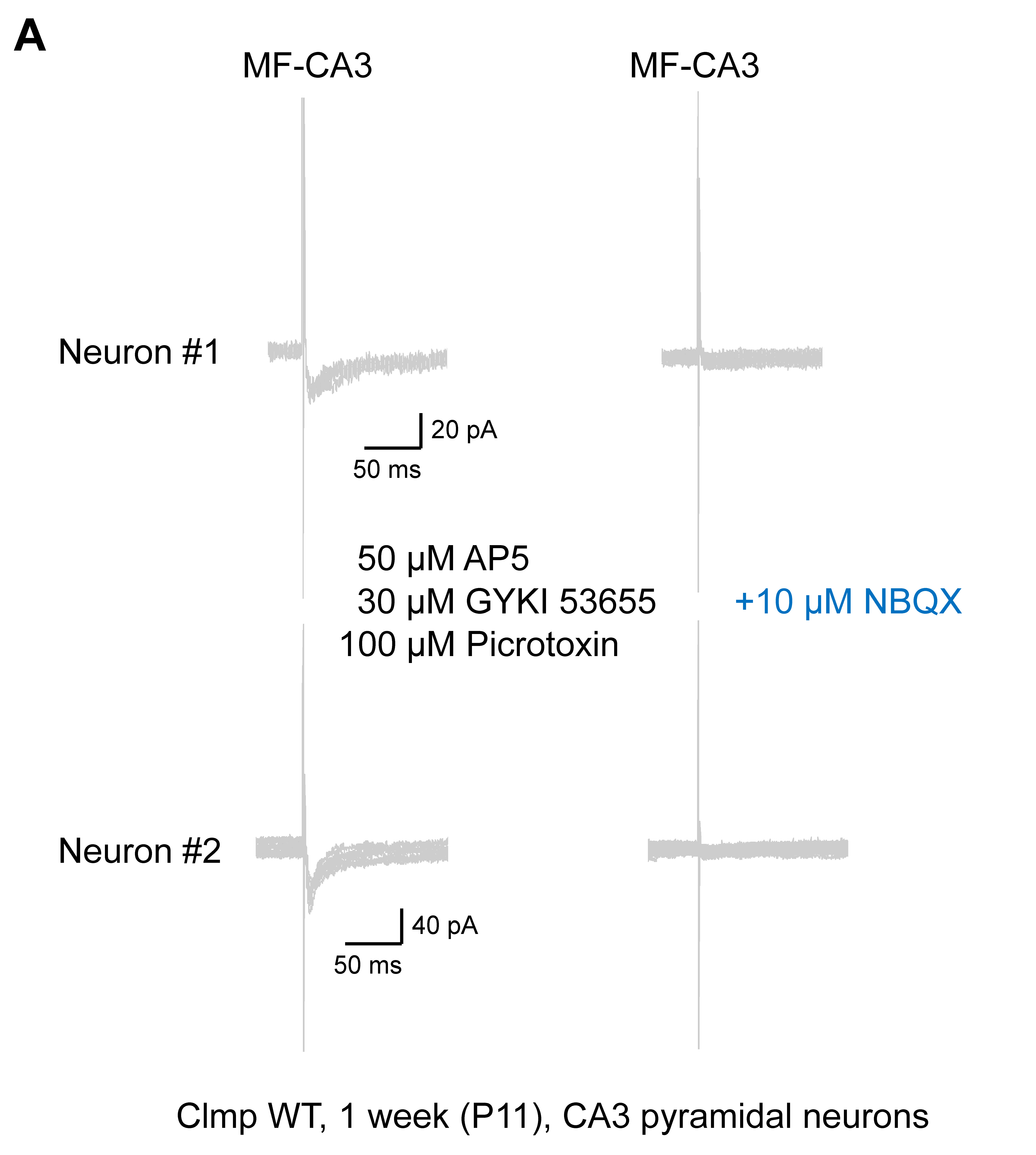

Supplement: Supplementary Figure 4 — Elimination of KAR eEPSCs by NBQX at MF-CA3 synapses. (A) KAR eEPSCs isolated by the treatment of the slices with GYKI 53655 (AMPAR antagonist), AP5 (NMDAR antagonist), and picrotoxin (GABA receptor antagonist) were eliminated by the treatment of NBQX (AMPAR and KAR antagonist), as shown by two examples of KAR eEPSCs recorded at WT MF-CA3 synapses (week 1). [file Image_4.JPEG]
